# Host metabolites stimulate the bacterial proton motive force to enhance the activity of aminoglycoside antibiotics

**DOI:** 10.1371/journal.ppat.1007697

**Published:** 2019-04-29

**Authors:** Aurélie Crabbé, Lisa Ostyn, Sorien Staelens, Charlotte Rigauts, Martijn Risseeuw, Maarten Dhaenens, Simon Daled, Heleen Van Acker, Dieter Deforce, Serge Van Calenbergh, Tom Coenye

**Affiliations:** 1 Laboratory of Pharmaceutical Microbiology, Ghent University, Ghent, Belgium; 2 Laboratory for Medicinal Chemistry, Ghent University, Ghent, Belgium; 3 ProGenTomics, Laboratory of Pharmaceutical Biotechnology, Ghent University, Ghent, Belgium; Emory University School of Medicine, UNITED STATES

## Abstract

Antibiotic susceptibility of bacterial pathogens is typically evaluated using *in vitro* assays that do not consider the complex host microenvironment. This may help explaining a significant discrepancy between antibiotic efficacy *in vitro* and *in vivo*, with some antibiotics being effective *in vitro* but not *in vivo* or vice versa. Nevertheless, it is well-known that antibiotic susceptibility of bacteria is driven by environmental factors. Lung epithelial cells enhance the activity of aminoglycoside antibiotics against the opportunistic pathogen *Pseudomonas aeruginosa*, yet the mechanism behind is unknown. The present study addresses this gap and provides mechanistic understanding on how lung epithelial cells stimulate aminoglycoside activity. To investigate the influence of the local host microenvironment on antibiotic activity, an *in vivo*-like three-dimensional (3-D) lung epithelial cell model was used. We report that conditioned medium of 3-D lung cells, containing secreted but not cellular components, potentiated the bactericidal activity of aminoglycosides against *P*. *aeruginosa*, including resistant clinical isolates, and several other pathogens. In contrast, conditioned medium obtained from the same cell type, but grown as conventional (2-D) monolayers did not influence antibiotic efficacy. We found that 3-D lung cells secreted endogenous metabolites (including succinate and glutamate) that enhanced aminoglycoside activity, and provide evidence that bacterial pyruvate metabolism is linked to the observed potentiation of antimicrobial activity. Biochemical and phenotypic assays indicated that 3-D cell conditioned medium stimulated the proton motive force (PMF), resulting in increased bacterial intracellular pH. The latter stimulated antibiotic uptake, as determined using fluorescently labelled tobramycin in combination with flow cytometry analysis. Our findings reveal a cross-talk between host and bacterial metabolic pathways, that influence downstream activity of antibiotics. Understanding the underlying basis of the discrepancy between the activity of antibiotics *in vitro* and *in vivo* may lead to improved diagnostic approaches and pave the way towards novel means to stimulate antibiotic activity.

## Author summary

There is a poor correlation between the activity of antibiotics in the laboratory and in patients, including in several infectious diseases of the respiratory tract. What may help explaining differences between antibiotic activity *in vitro* and *in vivo* is that current antibiotic susceptibility tests do not consider the *in vivo* lung environment. The lung environment contains many factors that may influence bacterial susceptibility to antibiotics. This includes lung epithelial cells, which have been shown to improve the activity of aminoglycoside antibiotics. Yet, how lung epithelial cells increase aminoglycoside activity is currently unknown. Here, we cultured lung epithelial cells in an *in vivo*-like model and found that they secrete metabolites that enhance the activity of aminoglycoside antibiotics. We found that host cell secretions increased antibiotic uptake through stimulation of bacterial metabolism, which in turn resulted in enhanced activity. Our findings highlight that cross-talk between host and bacterial metabolisms contributes to the efficacy of antibiotic treatment. Understanding how the host metabolism influences antibiotic activity may open up therapeutic avenues to exploit host metabolism for improving antibiotic activity and help explaining discrepancies between antibiotic efficacy *in vitro* and *in vivo*.

## Introduction

While many biochemical substances that modulate antibiotic activity are known [1–10], the influence of the local environment at the host-pathogen interface on bacterial responses to antibiotics is still poorly understood [4]. Supplementation of exogenous metabolites has been found to enhance the activity of certain antibiotics, including aminoglycosides. Combining various carbon sources, such as carbohydrates (e.g. glucose, fructose), amino acids (e.g. alanine), and metabolites from the tricarboxylic acid cycle (e.g. pyruvate, citrate) with aminoglycoside antibiotics rendered antibiotic resistant bacteria susceptible again, eradicated persister cells, and/or reduced biofilm viability [1,2,6–8]. Hence, metabolic adjuvants have raised excitement for therapeutic applications against multi-drug resistant bacteria. Yet, whether and how the complex host metabolic environment influences antibiotic activity is not fully understood. These insights are important to evaluate the potential role of the host in the clearance of bacterial pathogens during antibiotic treatment of an infection, and may help opening novel avenues to improve the correlation between antibiotic susceptibility profiles *in vitro* and *in vivo*. This is particularly relevant for infectious diseases for which antibiotic therapy chosen based on susceptibility assays frequently does not lead to clinical improvement [11–14], including in respiratory tract infections in people with cystic fibrosis [11]. Indeed, these studies denote that some antimicrobial agents are effective *in vitro* but not *in vivo* or vice versa. We and others previously demonstrated that lung epithelial cells modulate the activity of antibiotics *in vitro* [15–19]. In particular, culturing biofilms of *Pseudomonas aeruginosa* on the surface of *in vivo*-like three-dimensional (3-D) lung epithelial cells enhanced the activity of aminoglycosides as compared to when these same biofilms were cultured on a plastic surface [19]. Furthermore, Wu et al. recently demonstrated that *P*. *aeruginosa* isolated directly from mouse lungs was more susceptible to aminoglycosides as compared to laboratory-grown cultures, suggesting that host factors influence antibiotic activity *in vivo* [15]. In the present study we aimed at elucidating (i) whether the host microenvironment influences aminoglycoside activity against bacterial pathogens, (ii) if host metabolites can potentiate aminoglycoside activity, and (iii) what the underlying mode of action is. To this end, we assessed antibiotic activity against biofilms formed in the presence of conditioned medium of 3-D lung epithelial cells. The 3-D lung epithelial cell model has repeatedly been shown to better mimic physiological characteristics of *in vivo* lung epithelium as compared to conventional 2-D monolayers, especially relating to mucosal immunity (barrier function, polarity, mucus production, and cytokine production) [19–23]. We found that 3-D cell secretions potentiated the activity of aminoglycosides against several bacterial species. In contrast, secretions of the same lung epithelial cell type grown as monolayers did not influence antibiotic activity. Next, we revealed the mechanistic basis of the potentiating activity of 3-D cell secretions, by demonstrating a stimulation of the bacterial PMF by host cell factors. Finally, our data suggest that host metabolites improved activity of the aminoglycoside tobramycin. These findings highlight a significant contribution of the host metabolism to the activity of antibiotic treatment.

## Results

### The microenvironment of 3-D but not 2-D lung epithelial cells potentiates aminoglycoside activity

**Fig 1:**
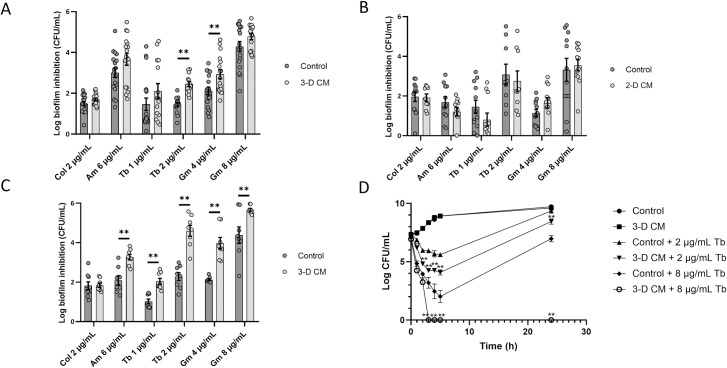
Antibiotic activity against *P*. *aeruginosa* PAO1 in conditioned medium derived from 3-D and 2-D lung epithelial cells. Biofilm inhibition by different antibiotics was determined after 4h of incubation in the presence of (A) 3-D CM derived from 10^6^ cells/mL, (B) 2-D CM derived from 10^6^ cells/mL, and (C) 3-D CM derived from 4 x 10^6^ cells/mL. (D) Time-kill curve using different concentrations of tobramycin in the presence of 3-D CM (derived from 4 x 10^6^ cells/mL) or control medium. Control medium was GTSF-2. Col = colistin, Am = amikacin, Tb = tobramycin, Gm = gentamicin. * p < 0.05, ** p < 0.01, n ≥ 3. Error bars represent standard error of the mean.

To assess if the host microenvironment influences the activity of antibiotics, the ability of antibiotics to inhibit biofilm formation for 4h was evaluated in conditioned medium of *in vivo*-like 3-D lung epithelial cells (3-D CM) and control medium (GTSF-2). 3-D CM was obtained by incubating differentiated 3-D A549 cells with fresh medium for 2h (1 x 10^6^ cells/mL), and collecting the non-cellular medium fraction. These tests were initially performed with *P*. *aeruginosa* PAO1, a well-studied biofilm-forming strain. 3-D CM increased the activity of gentamicin and tobramycin, and the level of potentiation varied with the antibiotic and concentration used, while the activity of colistin was not influenced by 3-D CM (**Fig 1A**). In contrast, CM from A549 monolayers (2-D CM) grown at the same cell density as the 3-D CM (1 x 10^6^ cells/mL) did not significantly influence the activity of any of the tested antibiotics (**Fig 1B**). We subsequently investigated whether increasing the 3-D lung epithelial cell density further enhanced the observed changes in antibiotic activity. CM obtained from a higher cell density (4 x 10^6^ cells/mL) strongly potentiated all tested aminoglycosides, while leaving the activity of colistin unchanged (**Fig 1C**). For all subsequent experiments, 3-D CM from high cell density (4 x 10^6^ cells/mL) cultures was used. 3-D CM did not influence the activity of antibiotics against established biofilms of *P*. *aeruginosa* PAO1 (**S1 Fig**). We observed a minor effect of 3-D CM on the MIC of tobramycin (2-fold decrease), and a 4-fold decrease in the MIC of colistin (**S1 Table**). Time-kill curves determined using a tobramycin concentration of 2 μg/mL and 8 μg/mL (2x and 8x MIC, respectively) revealed a significantly increased bactericidal activity in the presence of 3-D CM (**Fig 1D**). At 8 μg/mL tobramycin, bacterial regrowth was observed at the 24h time point for the control medium, while no culturable cells were detected (i.e. <10^2^ CFU/mL) in the presence of 3-D CM (p < 0.01).

### The potentiating effect of 3-D cell conditioned medium towards aminoglycosides is observed for most other *P*. *aeruginosa* strains and *Pseudomonas* species, and for few other bacterial species

Next, we evaluated whether the potentiating effect of 3-D CM is strain, genus or species-dependent. The majority of *P*. *aeruginosa* cystic fibrosis clinical and environmental isolates tested showed enhanced susceptibility to tobramycin or gentamicin (the latter antibiotic was only used when ≤ 1 log biofilm inhibition was observed at ≥ 256 μg/mL tobramycin) in the presence of 3-D CM (10/12 tested, 83.3%) (**Table 1**). Only for strains DK2 and LES400 no significant potentiating effect could be observed. For all tobramycin resistant strains tested (1709–12, Mi126, AMT0023-34) increased susceptibility to tobramycin was observed in the presence of 3-D CM. All non-*aeruginosa Pseudomonas* species tested (*P*. *fluorescens*, *P*. *putida*, *P*. *stutzeri*) also exhibited a decreased tolerance to tobramycin in the presence of 3-D CM. The potentiating effect was also observed in a limited number of other bacterial species (3/10 tested, 30.0%), i.e. *Staphylococcus aureus*, *Enterococcus faecium* and *Salmonella typhimurium*. In contrast, the activity of aminoglycosides was inhibited by 3-D CM for *Acinetobacter baumannii*, *Escherichia coli*, and *Achromobacter xylosoxidans* (**Table 1**).

**Table 1:** Potentiating effect of 3-D CM against biofilm formation of various bacterial species. Strains for which a potentiating effect of aminoglycosides was observed are highlighted in green, those for which the antibiotic activity was unchanged between 3-D CM and control are labelled in yellow, and the strains for which aminoglycoside activity was inhibited by 3-D CM are coloured in red. The standard error of the mean is indicated.

Strain	Fold-change antibiotic potentiation (3-D CM / control)	Antibiotic and concentration tested
***P*. *aeruginosa* PAO1**	606,0 ± 286,8**	Tobramycin (2 μg/mL)
*P*. *aeruginosa* AA44	225.9 ± 129.4*	Tobramycin (5 μg/mL)
*P*. *aeruginosa* AA2	79.2 ± 44.8**	Tobramycin (8 μg/mL)
*P*. *aeruginosa* **AA43**	407.3 ± 123.4**	Tobramycin (8 μg/mL)
*P*. *aeruginosa* LESB58	15.5 ± 4.4*	Tobramycin (32 μg/mL)
*P*. *aeruginosa* LES400	2.8 ± 1.6	Tobramycin (32 μg/mL)
*P*. *aeruginosa* DK2	7.9 ± 4.3	Tobramycin (0.5 μg/mL)
*P*. *aeruginosa* 1709–12	386.8 ± 280.1**	Tobramycin (2 μg/mL)
*P*. *aeruginosa* Mi126	7053.5 ± 6681.9**	Tobramycin (256 μg/mL)
*P*. *aeruginosa* AMT0023-34	1487.6 ± 898.2*	Tobramycin (192 μg/mL)
*P*. *aeruginosa* Jpn1563	209.8 ± 58.1**	Tobramycin (2 μg/mL)
*P*. *aeruginosa* Pr335	39.6 ± 10.3**	Tobramycin (1 μg/mL)
*P*. *putida* KT2440	286.3 ± 267.6*	Tobramycin (1 μg/mL)
*P*. *fluorescens*	181.4 ± 95.8**	Tobramycin (0.5 μg/mL)
*P*. *stutzeri*	94.5 ± 51.8*	Tobramycin (0.5 μg/mL)
*Staphylococcus aureus* SP123	58.8 ± 47.1*	Tobramycin (2 μg/mL)
*Enterococcus facium*	7.8 ± 2.4**	Gentamicin (8 μg/mL)
*Salmonella typhimurium*	5.1 ± 1.3**	Tobramycin (4 μg/mL)
*Acinetobacter baumannii* AB5075	0.5 ± 0.1*	Tobramycin (2 μg/mL)
*Achromobacter xylosoxidans*	0.1 ± 0.02**	Gentamicin (256 μg/mL)
*Burkholderia cenocepacia* K56-2	28.5 ± 15.9	Tobramycin (156 μg/mL)
*Escherichia coli*	0.1 ± 0.07**	Tobramycin (0.5 μg/mL)
*Streptococcus anginosus*	1.0 ± 0.4	Tobramycin (256 μg/mL)
*Rothia mucilaginosa*	1.4 ± 0.4	Tobramycin (64 μg/mL)
*Gemella haemolysans*	0.8 ± 0.1	Tobramycin (2 μg/mL)

* p ≤ 0.05,

** p < 0.01

### Tobramycin uptake in *P*. *aeruginosa* is increased by the 3-D cell conditioned medium

**Fig 2:**
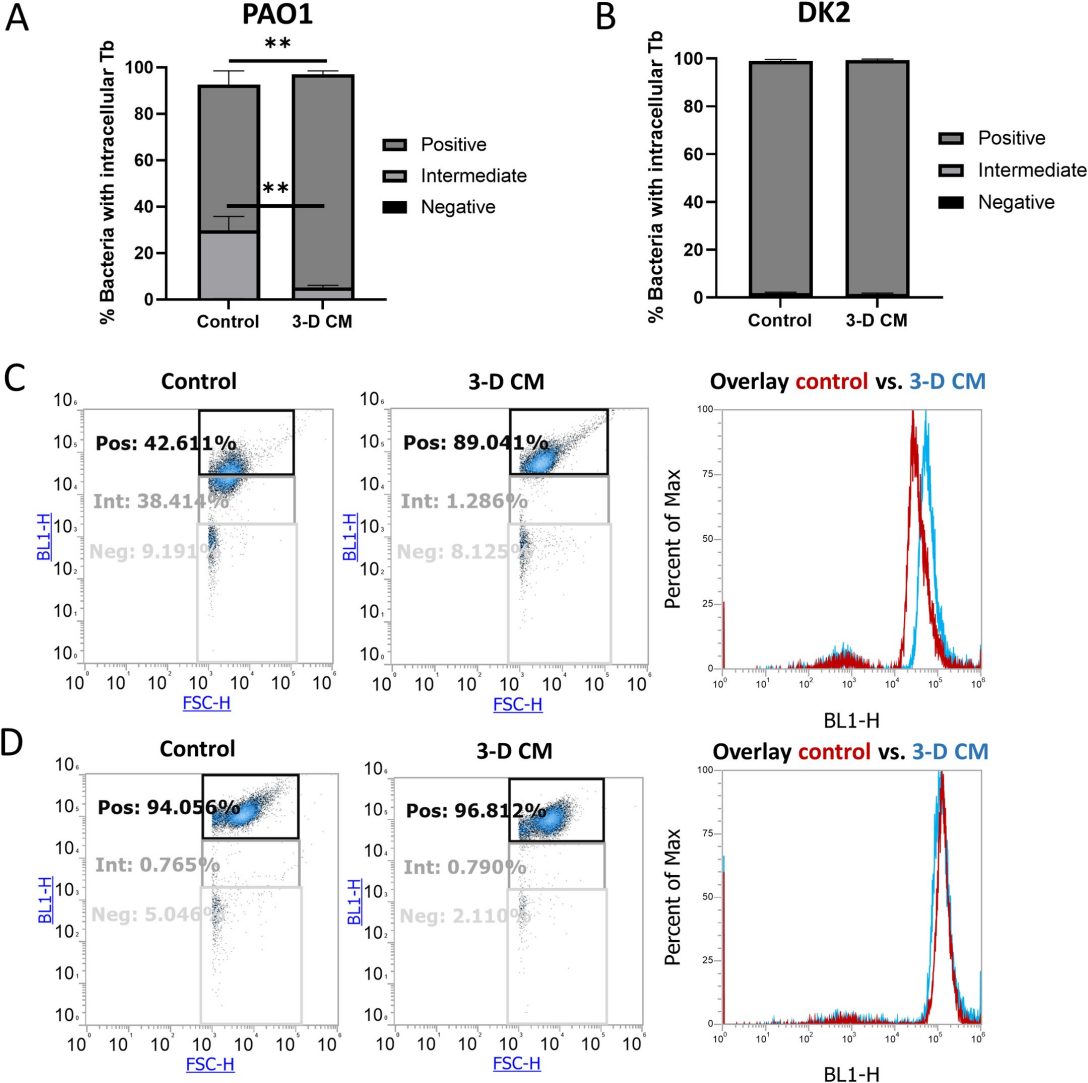
Uptake of BODIPY-tobramycin by *P*. *aeruginosa* PAO1 (A, C) and DK2 (B, D) in 3-D CM versus control medium, determined using flow cytometry analysis. Tobramycin uptake was assessed based on the fraction of the population that fell into three respective gates: negative, intermediate and positive. Negative and positive gates were determined respectively using a negative control (untreated sample) and BODIPY-tobramycin concentrations that resulted in maximal fluorescence intensity (S2 Fig). Bacteria whose fluorescence was situated between the positive and negative gates represent the intermediate population. Biofilms were formed for 4h in the presence of 0.75 μg/mL BODIPY-tobramycin. Panels A and B are derived from the dot plot graphs of each replicate (left and middle image) in panels C and D, respectively (forward scatter signal in X-axis, fluorescence intensity in Y-axis). Panels C and D show one representative replicate. The right image of panels C and D present an overlay of the histograms from control and 3-D CM samples, showing the fluorescence intensity on the X-axis and the percentage of the analysed cell population in the Y-axis. Control medium was GTSF-2. ** p < 0.01, n ≥ 3. Error bars represent standard error of the mean.

To elucidate the mode of action behind the potentiating activity of 3-D CM, we evaluated whether intracellular tobramycin levels were increased upon exposure to 3-D CM, using fluorescent BODIPY-labelled tobramycin and flow cytometry. Flow cytometry settings and gates were determined using negative and positive controls, wherein the fraction of the bacterial population that did not contain detectable levels of BODIPY-tobramycin was set to be located in the “negative” gate, the fraction that was situated in the “positive” gate was saturated for BODIPY-tobramycin, and the fraction in between the positive and negative gates, was labelled as the “intermediate” gate (**S2 Fig**). A concentration of 0.5 μg/mL or 0.75 μg/mL BODIPY-tobramycin was used in further experiments as these concentrations resulted in a partially saturated population under control conditions, hereby enabling to observe potential increases in BODIPY-tobramycin uptake upon exposure to 3-D CM (**S2 Fig**). 3-D CM enhanced the fraction of positive cells (p < 0.01 for 0.5 and 0.75 μg/mL BODIPY-tobramycin), while lowering the fraction of intermediate cells (p < 0.05 for 0.5 μg/mL and p < 0.01 for 0.75 μg/mL BODIPY-tobramycin) which is due to an increase in mean fluorescence intensity of the whole population (**Figs 2A and 2C and S3A**).

We also tested one of the few *P*. *aeruginosa* strains that showed a small (non-significant) enhanced susceptibility to tobramycin in the presence of 3-D CM, i.e. strain DK2. This strain had the same MIC for tobramycin in the control medium as PAO1 (**S1 Table**). The majority of *P*. *aeruginosa* DK2 cells exposed to BODIPY-tobramycin in control medium was already positive (saturated) for BODIPY-tobramycin at a concentration of 0.75 μg/mL, and no difference between control medium and 3-D CM could be observed (p > 0.05) (**Fig 2B and 2D**). A lower concentration of BODIPY-tobramycin (0.5 μg/mL) resulted in a partially saturated *P*. *aeruginosa* DK2 population in control medium, and 3-D CM induced a smaller increase in tobramycin uptake (1.9 ± 0.2-fold) compared to *P*. *aeruginosa* PAO1 tested at the same concentration (4.9 ± 1.4-fold) (p < 0.05) (**S3B Fig**). These results are in line with the biofilm inhibition data, where 3-D CM exerted a stronger potentiating effect in PAO1 (606.0 ± 286.8-fold) compared to DK2 (7.9 ± 4.3-fold) (p < 0.01).

**Fig 3:**
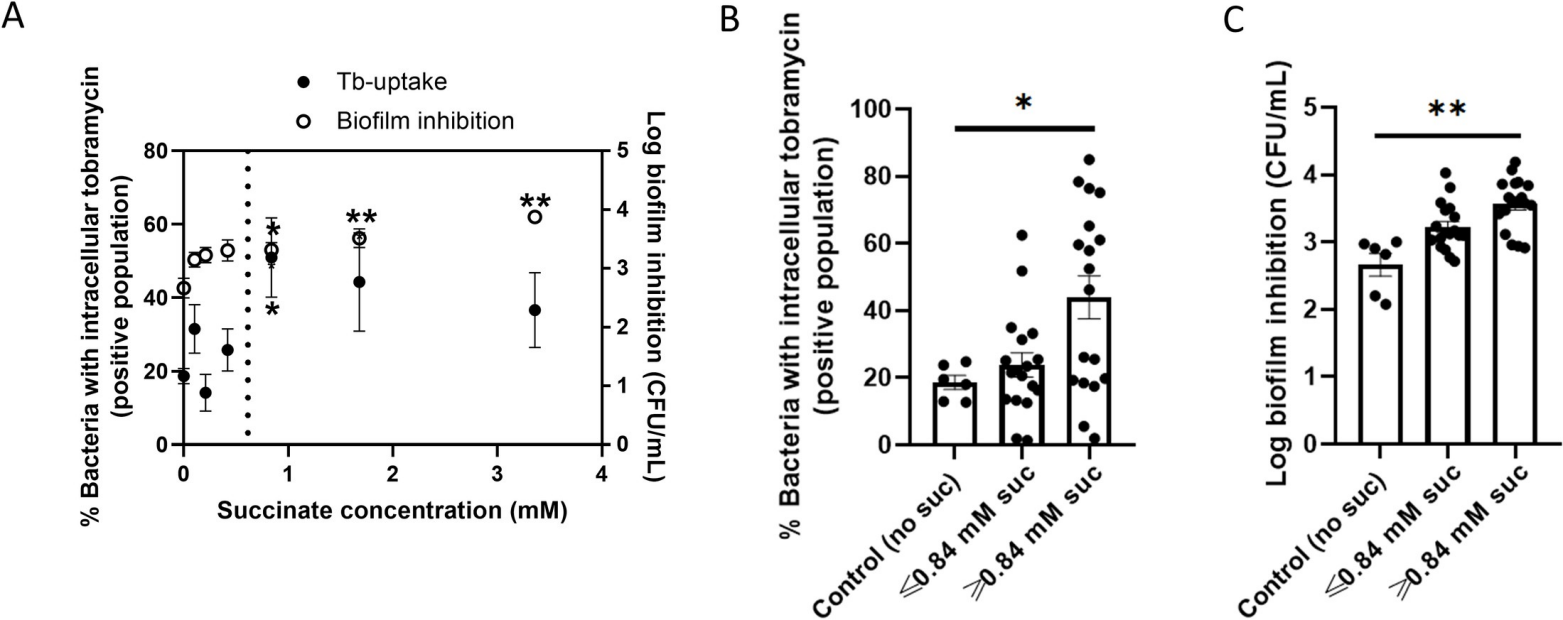
Correlation of tobramycin uptake and biofilm inhibition using the tobramycin-potentiator succinate. (A) Biofilm inhibition of *P*. *aeruginosa* PAO1 by 2 μg/mL tobramycin and uptake of BODIPY-tobramycin in control medium supplemented with a two-fold dilution series of succinate (0.11–3.36 mM). For BODIPY-tobramycin uptake, flow cytometry analysis was performed, the positive population is presented. Biofilms were formed for 4h. A threshold succinate concentration was observed (0.84 mM) above which both potentiation of biofilm inhibition and an increase in tobramycin uptake occurred (dotted line). This threshold was used for defining the groups in panels (B) and (C). Control medium was GTSF-2, suc = succinate. *p ≤ 0.05 ** p < 0.01, n ≥ 3. Error bars represent standard error of the mean.

To further assess whether tobramycin uptake was correlated with inhibition of biofilm formation, we used a standard series of the previously described tobramycin-potentiator succinate [7]. We observed that at concentrations ≥ 0.84 mM succinate, both tobramycin activity and uptake were significantly enhanced by this molecule (p ≤ 0.05) (**Fig 3**).

### Tobramycin potentiation by 3-D cell conditioned medium is dependent on the PMF

**Fig 4:**
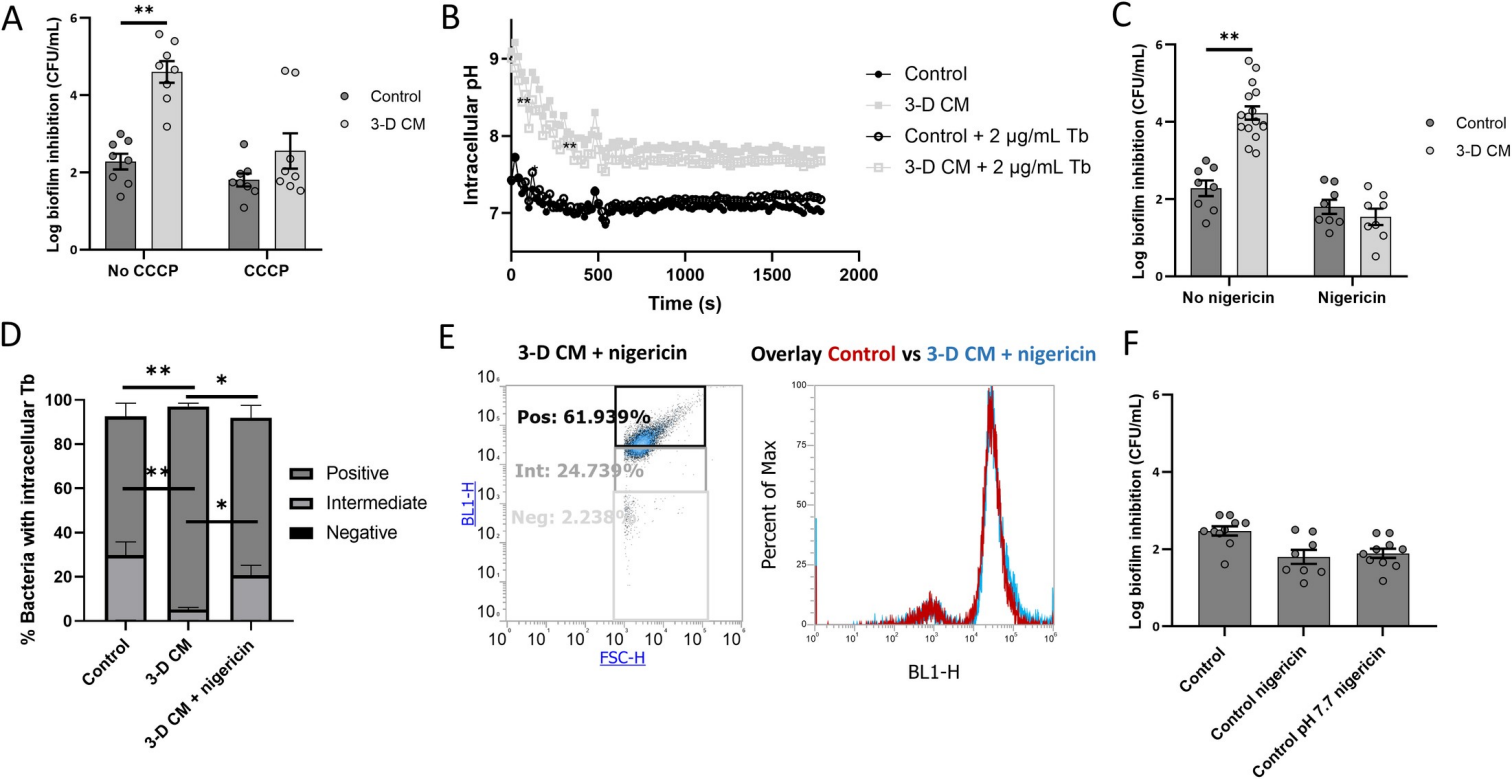
Evaluating the role of the proton motive force on the potentiating effect of 3-D CM on *P*. *aeruginosa* PAO1. (A) Biofilm inhibition by 2 μg/mL tobramycin in 3-D CM and control medium in the presence or absence of 100 μM of the proton motive force disruptor CCCP. (B) Intracellular pH of *P*. *aeruginosa* PAO1 in the presence of 3-D CM or control medium with or without tobramycin treatment (2 μg/mL, monitored every 20 s) (representative replicate). (C) Biofilm inhibition by 2 μg/mL tobramycin in 3-D CM and control medium in the presence or absence of the potassium ionophore nigericin (10 μM) and excess levels of potassium (150 mM). (D) Uptake of BODIPY-tobramycin (0.75 μg/mL) by *P*. *aeruginosa* PAO1 in control medium, and 3-D CM in the presence or absence of nigericin/excess K^+^. Panel D is derived from the dot plot graph in panel E (left) (representative replicate) and Fig 2 panel C (forward scatter signal in X-axis, fluorescence intensity in Y-axis). The right image of panel E presents an overlay of the histograms from control and 3-D CM + nigericin/excess K^+^ samples, showing the fluorescence intensity on the X-axis and the percentage of the analysed cell population in the Y-axis. (F) Biofilm inhibition by 2 μg/mL tobramycin in control medium with increased pH (7.7) and nigericin to equalize intra- and extracellular pH to higher pH. Tb = tobramycin. Control medium was GTSF-2. * p < 0.05, ** p < 0.01, n ≥ 3. Error bars represent standard error of the mean.

Higher intracellular levels of tobramycin could be due to higher uptake and/or lower efflux in response to 3-D CM. The uptake of aminoglycosides occurs in three phases: an energy-independent phase (ionic binding and/or diffusion through outer membrane porins) and two energy dependent phases (EDPI and EDPII) [24–26]. In order to find out which phase(s) of aminoglycoside uptake were influenced by 3-D CM, biofilm inhibition by tobramycin was studied in the presence of the proton ionophore CCCP, which enables inward transport of H^+^ across lipid membranes [27], hereby dissipating the proton motive force (PMF). The potentiating activity of 3-D CM was completely abolished by 100 μM CCCP (p > 0.05), while CCCP had no effect on tobramycin activity in control medium (**Fig 4A**).

To further elucidate the role of the bacterial PMF in the observed potentiating effect of 3-D CM, we determined intracellular pH levels using 2,7 -bis(2-carboxyethyl)-5(6)-carboxyfluorescein acetoxymethyl (BCECF-AM). As a difference in pH was observed between 3-D CM (pH 6.99 ± 0.28) and control medium (pH 7.29 ± 0.10), the pH of 3-D CM was increased to the control levels prior to determining the intracellular pH. The potentiating effect of 3-D CM was not affected by normalizing the pH between 3-D CM and control medium (**S5 Fig**).

We observed a consistent increase in intracellular pH of *P*. *aeruginosa* PAO1 in the presence of 3-D CM as compared to control medium, both in tobramycin treated (pH 7.7 for 3-D CM, pH 7.2 for control) and untreated samples (pH 7.7 for 3-D CM, pH 7.1 for control) (**Fig 4B**). To confirm that the high intracellular pH induced by 3-D CM is responsible for the enhanced tobramycin activity in the presence of 3-D CM, we inhibited the 3-D CM-induced increase in intracellular pH. To this end, we used the K^+^ ionophore nigericin in combination with excess levels of K^+^ to equalize intra- and extracellular pH levels through promotion of K^+^/H^+^ exchange, hereby preventing 3-D CM-induced increases in intracellular pH. The biofilm inhibition assay was repeated with 3-D CM in the presence of nigericin/excess K^+^, and this completely abolished the potentiating effect of 3-D CM (**Fig 4C**). Accordingly, the 3-D CM-induced increase in intracellular tobramycin levels could not be observed in the presence of nigericin/excess K^+^, and tobramycin activity of 3-D CM combined with nigericin/excess K^+^ equalled that of the control (**Figs 4D, 4E and 2C**). Finally, as pH affects tobramycin stability, we also assessed whether higher pH on its own could potentiate tobramycin. To this end, 1 M NaOH was used to increase the pH of the control medium to the level of the intracellular pH in 3-D CM (pH 7.7), where after intra- and extracellular pH levels were equalized using nigericin/excess K^+^. This approach did not result in tobramycin potentiation (**Fig 4F**).

We also determined the potentiating effect of 3-D CM in conditions that reduce bacterial metabolic activity by determining intracellular levels of tobramycin at 4°C. Also at 4°C, 3-D CM enhanced the fraction of bacteria that were positive for tobramycin, and lowered the fraction of intermediate and negative cells (**S4A Fig**). The main difference with the experiment at 37°C was the distinct presence of a negative, intermediate and positive population in the control medium, with an influence of 3-D CM observed in each of these subpopulations, without affecting the mean fluorescence intensity as observed in the histogram (**S4B Fig**).

### Efflux pumps and membrane permeability are not involved in the 3-D CM-mediated potentiation of tobramycin

**Fig 5:**
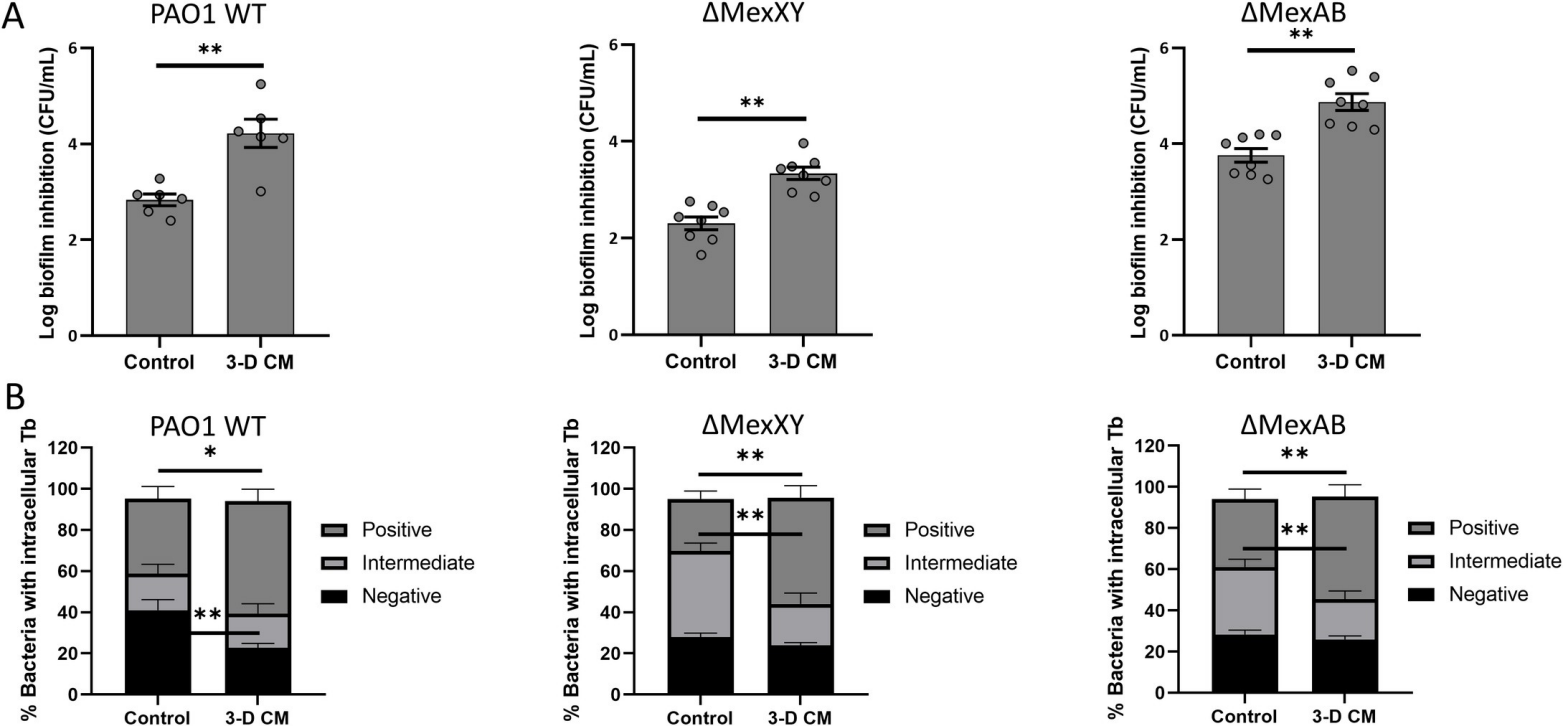
Biofilm inhibition (A) and BODIPY-tobramycin uptake (B) of efflux pump mutants *P*. *aeruginosa* ΔMexAB and ΔMexXY and isogenic wild type of *P*. *aeruginosa* PAO1 in the presence of 3-D CM or control medium. Biofilms were formed for 4h prior to sample processing, in the presence of a tobramycin concentration that resulted in at least 1 log biofilm inhibition (2 μg/mL for WT PAO1, 1 μg/mL for ΔMexXY and ΔMexAB). To determine the uptake of BODIPY-tobramycin using flow cytometry, a concentration of 0.75 μg/mL was used. Control medium was GTSF-2. Tb = tobramycin. * p < 0.05, ** p < 0.01, n ≥ 3. Error bars represent standard error of the mean.

We evaluated whether alternative mechanisms could explain the aminoglycoside potentiating activity of 3-D CM. Firstly, the role of efflux was evaluated by testing whether 3-D CM could potentiate tobramycin activity in mutants, in which known efflux pumps associated with the export of aminoglycosides in *P*. *aeruginosa* were inactivated, i.e. MexXY and MexAB [28,29]. Also in these mutants, 3-D CM potentiated the activity of tobramycin against biofilm formation and increased tobramycin uptake (**Fig 5**).

**Fig 6:**
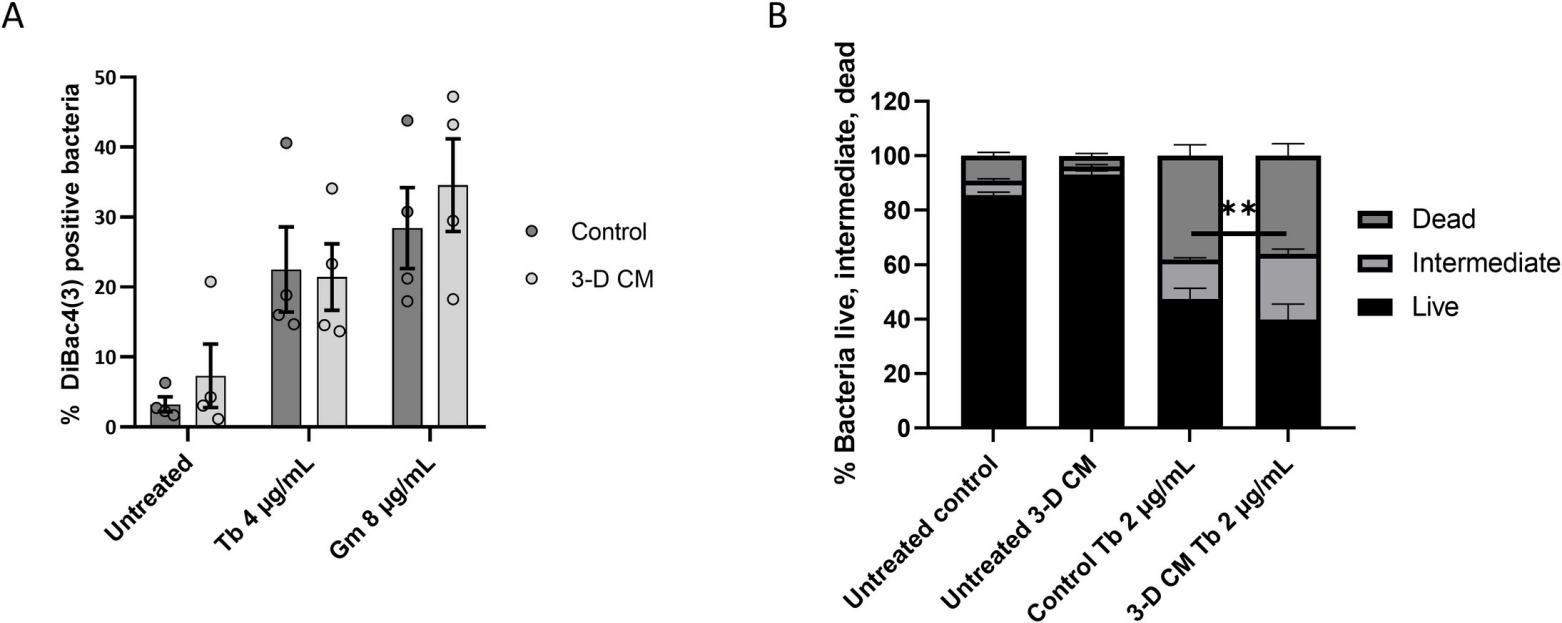
Influence of 3-D CM on membrane potential and permeability of *P*. *aeruginosa* PAO1. (A) DiBac4(3) assay. Samples were processed following 4h incubation in control medium or 3-D CM with antibiotic concentrations that caused membrane depolarization (4 μg/mL tobramycin, 8 μg/mL gentamicin). (B) Live/dead assay. The biofilm fraction was processed following 4h incubation in control medium or 3-D CM with or without 2 μg/mL tobramycin. Based on the level of SYTO9 and PI, three populations could be distinguished (live, intermediate, dead). Tb = tobramycin, Gm = gentamicin. ** p < 0.01, n ≥ 3. Error bars represent standard error of the mean.

Secondly, to understand whether the induced uptake of aminoglycosides by 3-D CM is due to changes in membrane permeability/potential, we used DiBac_4_(3). DiBac_4_(3) is a potential-sensitive dye that only enters depolarized cells, and can be used to evaluate changes in membrane potential, which in turn can be a result of increased membrane permeability. Exposure of *P*. *aeruginosa* PAO1 to tobramycin or gentamicin resulted in membrane depolarization at similar levels in both control medium or 3-D CM (**Fig 6A**). We also performed a SYTO9/PI assay (LIVE/DEAD); staining of bacteria with SYTO9/PI after antibiotic exposure typically results in three subpopulations: live, dead and intermediate [30]. The intermediate population has been reported to represent bacteria with enhanced inner and outer membrane permeability [30]. 3-D CM did not significantly alter the proportion of live and dead bacteria in the tobramycin-exposed biofilm, but a small increase in the intermediate population was observed (1.7-fold, p < 0.01) (**Fig 6B**).

### Host-produced metabolites potentiate aminoglycoside activity

**Fig 7:**
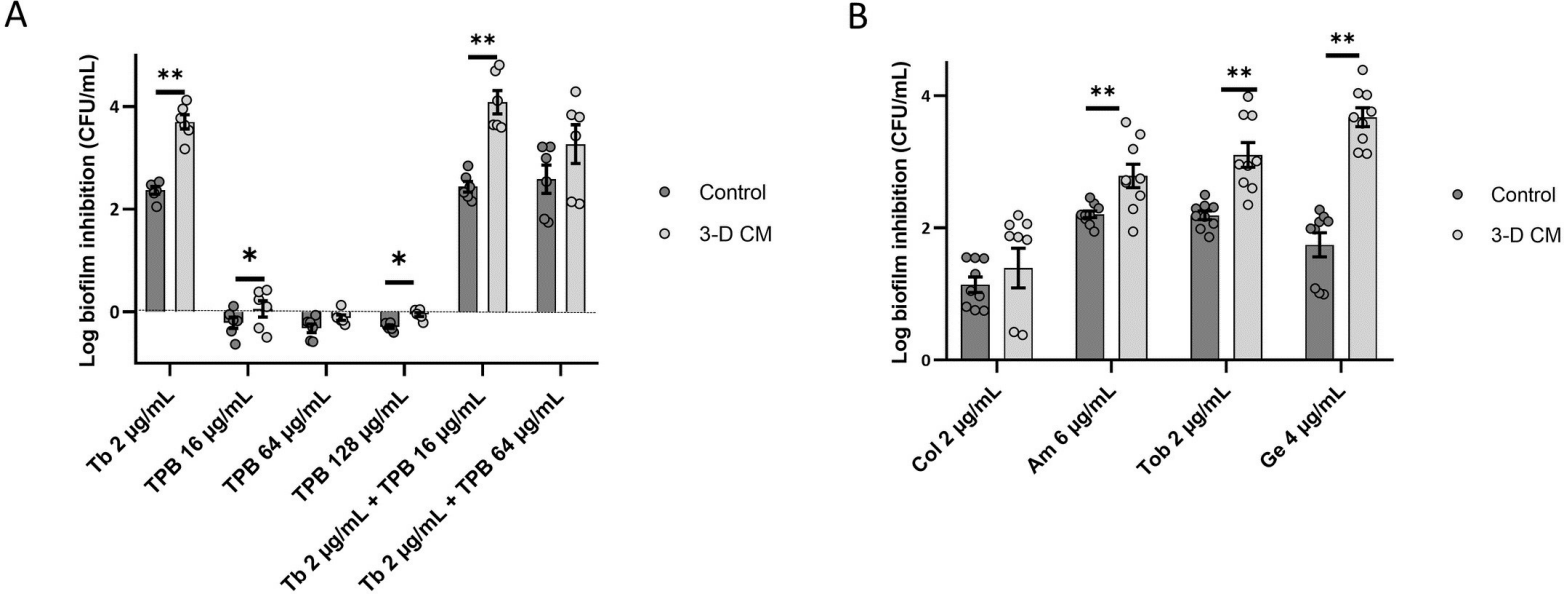
Role of host-produced metabolites in the potentiating effect of 3-D CM (A) Biofilm inhibition by 2 μg/mL tobramycin, triphenylbismuth dichloride and their combination. (B) Biofilm inhibition by 2 μg/mL tobramycin in control medium and 3-D CM filtered using a 3 kDa filter. Control medium was GTSF-2. ** p < 0.01, n ≥ 3. Error bars represent standard error of the mean.

Stimulating bacterial metabolism by addition of various carbohydrates (e.g. glucose, fructose) or derivatives of glycolysis/TCA cycle (e.g. pyruvate, succinate, glutamate, citrate) has been found to enhance the activity of aminoglycosides through promotion of the PMF [1,2,6–8]. To evaluate whether 3-D CM promoted the PMF by stimulating bacterial metabolism, we blocked a main metabolic pathway of *P*. *aeruginosa* by supplementing 3-D CM with the pyruvate dehydrogenase inhibitor triphenylbismuth dichloride (TPB) [31]. At 64 μg/mL TPB (i.e. 1/2 x MIC), the potentiating effect of 3-D CM was reduced (p > 0.05 between TPB-treated 3-D CM and control) (**Fig 7A**). However, lowering the TPB concentration to 16 μg/mL (1/8 x MIC) restored the effect of 3-D CM, indicating a concentration-dependent effect (**Fig 7A**).

To test whether host metabolites altered antibiotic activity, 3-D CM was filtered over a filter with a 3 kDa cut-off, and antibiotic-mediated biofilm inhibition in *P*. *aeruginosa* PAO1 was assessed. Potentiating activity for the three aminoglycosides tested was retained in the filtrate, which indicates that components that increase antibiotic activity are smaller than 3 kDa (**Fig 7B**).

**Fig 8:**
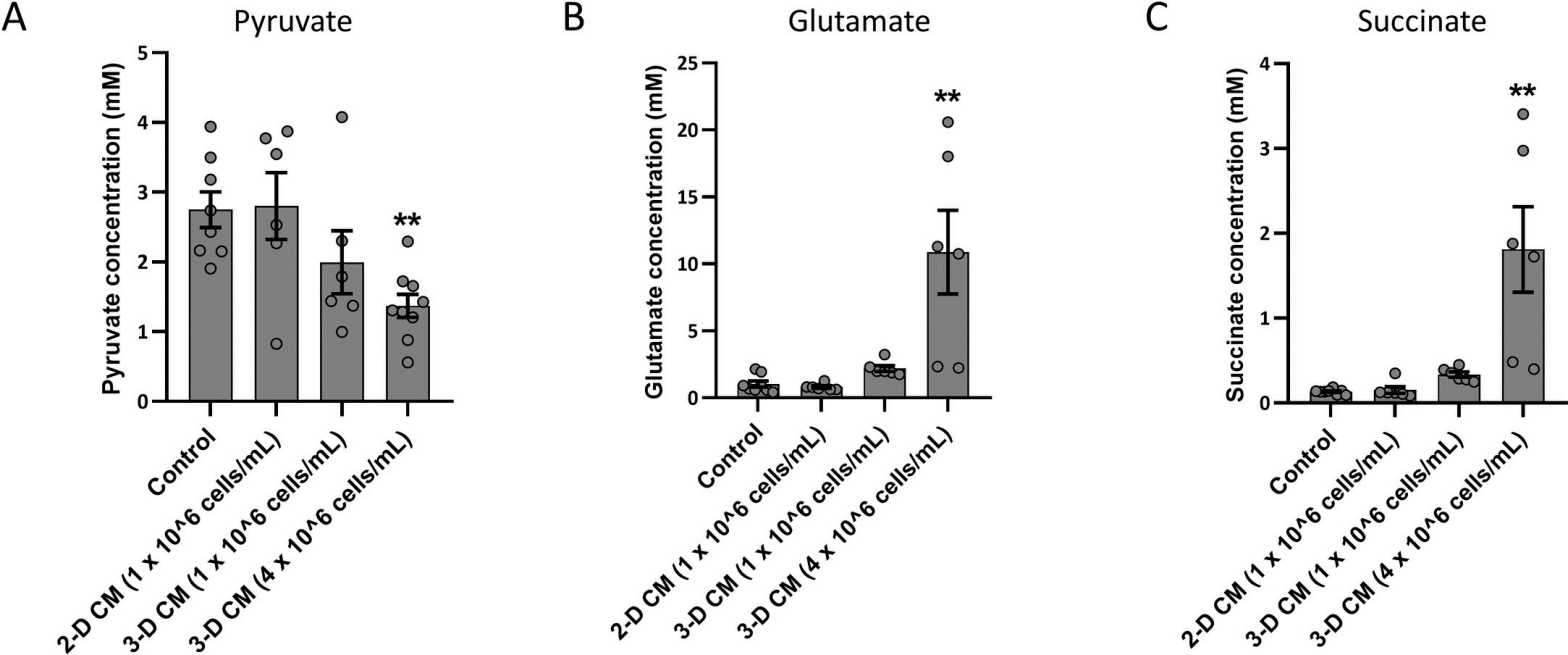
Quantification of pyruvate (A), succinate (B) and glutamate (C) in conditioned media of A549 lung epithelial cells (2-D CM derived from 1 x 10^6^ cells/mL, 3-D CM derived from 1 x 10^6^ cells/mL or 4 x 10^6^ cells/mL). Control medium was GTSF-2. ** p < 0.01, n ≥ 3. Error bars represent standard error of the mean.

**Fig 9:**
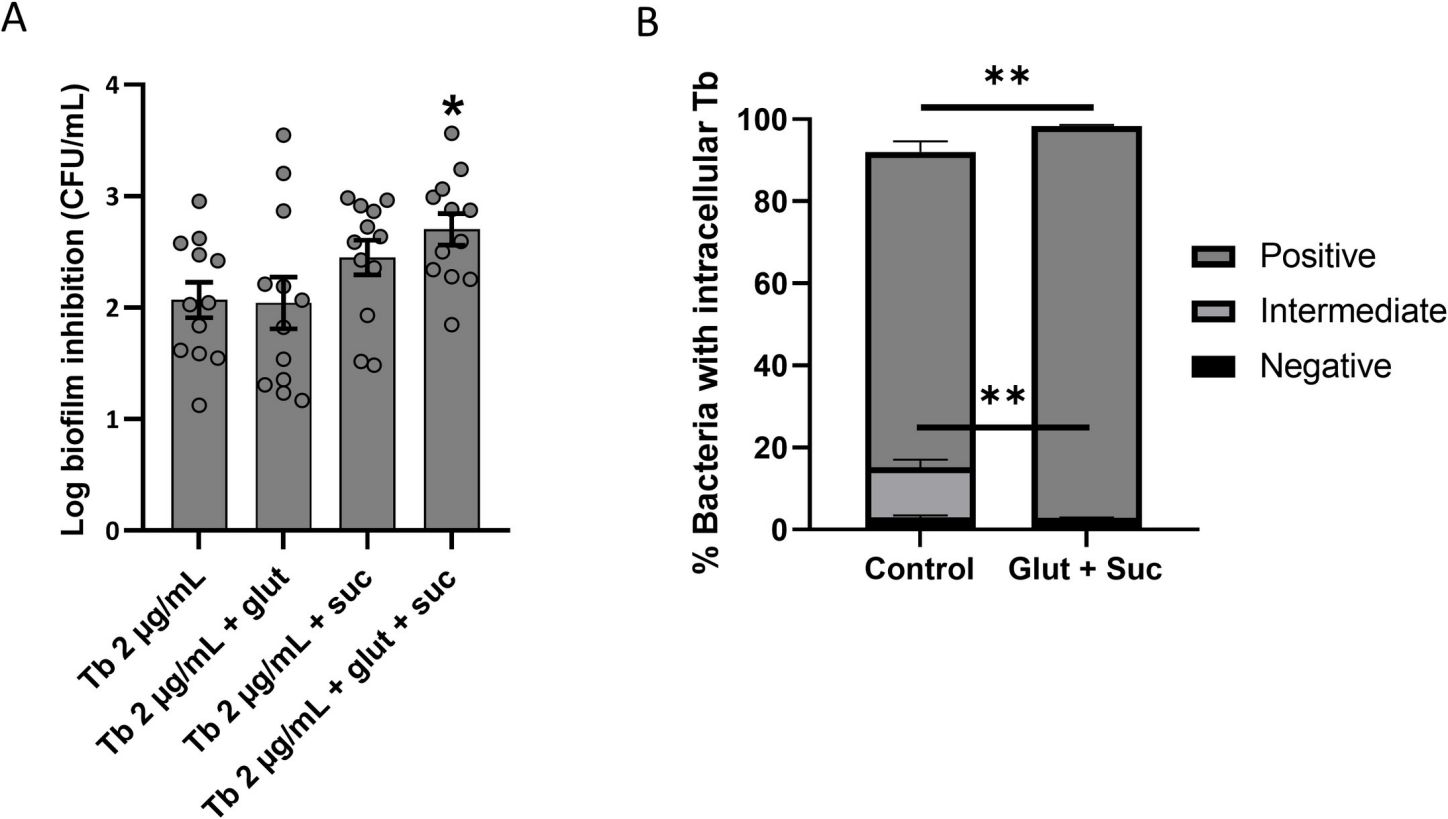
Influence of metabolites produced by 3-D lung epithelial cells on biofilm inhibition by tobramycin (A) and tobramycin uptake (B) by *P*. *aeruginosa* PAO1. Biofilm inhibition by tobramycin (2μg/mL) was tested in the absence or presence of glutamate (10.88 mM), succinate (1.81 mM) or their combination. Concentrations are determined based on quantification of these metabolites in 3-D CM derived from 4 x 10^6^ cells/mL. To determine the uptake of BODIPY-tobramycin using flow cytometry, a concentration of 0.75 μg/mL was used. Tb = tobramycin, glut = glutamate, suc = succinate. * p < 0.05, ** p < 0.01, n ≥ 3. Error bars represent standard error of the mean.

To determine which host metabolites potentiated aminoglycoside activity, we quantified various central metabolites (pyruvate, succinate and glutamate) in 3-D CM (derived from both 1 x 10^6^ cells/mL and 4 x 10^6^ cells/mL), 2-D CM (1 x 10^6^ cells/mL) and control. Control medium was found to contain 2.75 ± 0.26 mM pyruvate, and the pyruvate concentration was significantly lower in 3-D CM derived from a high cell number (1.37 ± 0.17 mM), indicating a consumption of approximately half of the pyruvate (**Fig 8A**). The concentration of pyruvate in 3-D CM derived from a low cell number was also decreased, but to a lesser extent and this decrease was not statistically significant. No significant differences were observed between control medium and 2-D CM indicating no significant consumption of pyruvate by 2-D cells. Similarly, both glutamate (10.88 ± 3.12 mM) and succinate (1.81 ± 0.51 mM) were significantly higher than control medium in 3-D CM derived from a high cell number (**Fig 8B and 8C**). However, 3-D CM derived from a low cell number contained higher levels of both glutamate and succinate than control and 2-D CM (low cell number), yet statistical significance was not reached. Next, we supplied control medium with glutamate, succinate, or both, at concentrations detected in 3-D CM (high cell number) and evaluated the activity of tobramycin and tobramycin uptake. The combination of glutamate and succinate significantly potentiated the biofilm inhibitory activity of tobramycin and tobramycin uptake, indicating that these metabolites are contributing factors to the observed potentiating effect of 3-D CM (**Fig 9**).

**Fig 10:**
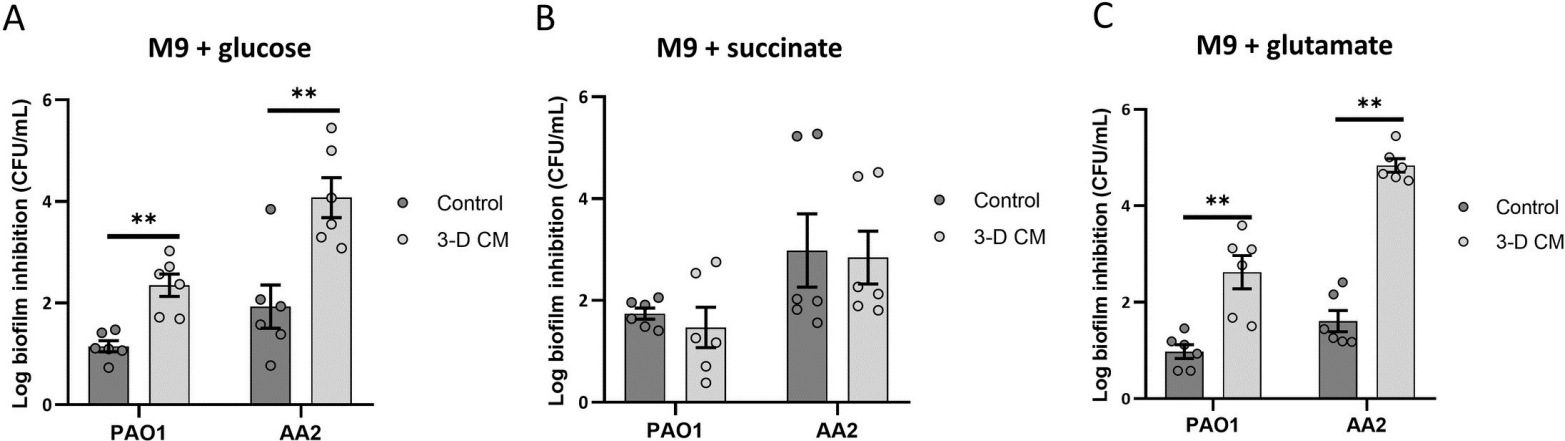
Biofilm inhibition of *P*. *aeruginosa* PAO1 or AA2 by tobramycin in the presence of 3-D CM or control medium after overnight culturing in M9 minimal medium supplemented with (A) glucose (10 mM), (B) succinate (15 mM), (C) glutamate (12 mM). For *P*. *aeruginosa* PAO1 and AA2, 2 μg/mL and 8 μg/mL tobramycin was used, respectively. Tb = tobramycin. ** p < 0.01, n ≥ 3. Error bars represent standard error of the mean.

Next, we evaluated whether the culture conditions prior to the biofilm inhibition experiment played a role in the potentiating activity of host metabolites. Instead of using rich growth medium (LB), overnight cultures were grown in M9 minimal medium supplemented with glucose, succinate or glutamate as sole carbon source, where after *P*. *aeruginosa* biofilm inhibition by tobramycin in control medium versus 3-D CM was evaluated. For both strains tested (PAO1, AA2), prior culturing in M9 glucose and M9 glutamate also resulted in enhanced tobramycin activity in the presence of 3-D CM (**Fig 10**). Interestingly, culturing of *P*. *aeruginosa* PAO1 or AA2 in M9 supplemented with succinate abolished the potentiating activity of 3-D CM. These findings indicate the culture conditions preceding antibiotic treatment influence the potentiating activity of host metabolites.

Finally, we ruled out a role for bicarbonate and host antimicrobial peptides in increasing aminoglycoside efficacy.

Antimicrobial peptides (e.g. β-defensins) and proteins (e.g. lysozyme) are produced by epithelial tissues and have been shown to exert synergistic effects when combined with several classes of antibiotics, including aminoglycosides [32]. Conservation of the potentiating effect in the 3 kDa filtrate rules out all antimicrobial proteins and many known antimicrobial peptides with a molecular mass > 3 kDa (e.g. CCL20, HBD-2) (**Fig 7B**). In addition, 3-D CM was treated with proteinase K or trypsin, where after the enzyme was removed by filtering over a 3 kDa filter and the activity of antibiotics to inhibit biofilm formation was assessed. Treatment of 3-D CM with proteinase K or trypsin did not affect the observed potentiating activity (**S6 Fig**), confirming that the potentiating compound is not proteinaceous. Bicarbonate was previously found to improve aminoglycoside activity through interference with the pH gradient [5], and to evaluate its role in the observed potentiating activity of 3-D CM, we tested whether 3-D CM could potentiate aminoglycosides in medium without bicarbonate. The potentiating activity of 3-D CM in the absence of bicarbonate was still observed (**S6 Fig**), but to a lesser extent compared to the medium that contained bicarbonate (p < 0.01).

## Discussion

During an infection, bacterial pathogens are exposed to a variety of host factors that influence the infection process and may affect their susceptibility to antimicrobial agents. These host factors are produced as part of normal tissue homeostasis (e.g. products of metabolism) or their production is triggered or enhanced upon sensing foreign microorganisms (e.g. defensins). While interactions between the host microenvironment and the pathogen are known to play a role in the establishment and persistence of an infection, there is limited knowledge on how the host microenvironment modulates the activity of antimicrobial agents. In the present study, we provide mechanistic insights on how lung epithelial cells modulate the activity of antibiotics, using an *in vivo*-like 3-D lung epithelial cell model. The 3-D organotypic lung model simulates key components of the lung mucosa that are important for the innate defence against microorganisms, including barrier function, apical-basolateral polarity, mucus production, and cytokine secretion [19–23]. We previously found that 3-D lung cells potentiated the activity of aminoglycosides [19]. In the present study, we were able to demonstrate that the potentiator effect was due to metabolites secreted by the 3-D lung cells, since effects by 3-D CM on aminoglycoside efficacy against biofilm inhibition were comparable to our previously reported effects by the 3-D lung cells. Strikingly, 3-D CM was able to strongly enhance the bactericidal activity of tobramycin, and even prevent bacterial regrowth in a time-kill assay. Host cell secretions resulted in an increase of intracellular tobramycin levels in the bacterial population, which was the result of an enhanced intracellular pH. An increase in intracellular pH leads to a higher ΔpH, which is an important component of the PMF. Since tobramycin uptake depends on the PMF [25], uptake of this antibiotic is promoted through a higher ΔpH. This is in line with recent reports where exogenous metabolites, including compounds derived from pyruvate metabolism (e.g. succinate, glutamate), were found to increase the activity of aminoglycosides by stimulating tricarboxylic acid cycle (TCA) activity and/or the phosphoenolpyruvate (PEP)-pyruvate-AcCoA pathway [2,6,7,33]. This in turn increased cellular respiration (through NADH production), PMF, antibiotic uptake and cell death. Inhibition of bacterial pyruvate dehydrogenase reduced the potentiating effect of 3-D CM, further supporting the involvement of the bacterial PMF. We examined whether host cells would secrete these previously reported metabolites at sufficient levels to influence tobramycin activity and found that 3-D cells consumed pyruvate and produced glutamate and succinate, which in turn led to tobramycin potentiation. These results suggest that host metabolites enhance tobramycin activity. Not only do our results fully support recent findings [2,7], they demonstrate that host cells secrete these antibiotic-potentiating metabolites to levels that can actually exert a biological effect in the complex microenvironment of the host. Nevertheless, we found that the culturing conditions preceding antibiotic treatment may influence the potentiating activity of these host metabolites. The observation that prior culturing in culture medium with a high concentration of the most potent metabolite (succinate) precluded potentiating activity of 3-D CM might imply that the PMF is already strongly promoted at the start of treatment. Hence, the maximal capacity of the PMF might be reached which would prevent additional stimulation by host metabolites.

Based on the Human Metabolome Database, concentrations of these metabolites *in vivo* are available for saliva (fluid from relevant mucosal tissue) and strongly vary within and between studies. For succinate, average concentrations of up to 2.3 mM (ranging from 0.06–4.5 mM) have been reported (http://www.hmdb.ca/metabolites/HMDB0000254), while for L-glutamate up to 13.6 ± 2.4 μM has been detected in saliva of healthy adults (http://www.hmdb.ca/metabolites/HMDB0000148). These levels approximate the *in vitro* concentration for succinate in our study, which was the metabolite contributing most to potentiation. In addition, the level of pyruvate present in the used cell culture medium (GTSF-2) of 2.75 ± 0.26 mM (corresponding to the concentration provided by the manufacturer of 3 mM) is also in the range of reported studies—up to 5.4 mM on average in saliva (ranging from 0.1–11 mM) (http://www.hmdb.ca/metabolites/HMDB0000243). Based on this information, aminoglycoside potentiation appears possible *in vivo*, but might strongly vary for different body sites and between individuals. Therefore, future experiments will be needed to determine individual metabolite concentrations at relevant mucosal tissue locations, and to link metabolite(s) presence/concentration with antibiotic activity. Nevertheless, while we used a model system that reflects important traits of the parental lung epithelium, it should be noted that the *in vivo* environment at the host-pathogen interface is more complex. Hence, additional factors (such as innate immune cells and other lung cells, paracrine signalling, oxygen levels, pH, and ionic content) might generate a different metabolic environment than represented in this study, leading to a differential response to antibiotics. Wu et al. (2017) recently found an increased aminoglycoside activity against *P*. *aeruginosa* when A549 lung epithelial cells and neutrophils were co-cultured as 2-D monolayers. Neutrophils were found to be the main contributor to the observed effect, among others due to production of reactive oxygen species (ROS). The observation that neutrophils and not A549 monolayer cells potentiated tobramycin is in line with our results, as we found that conditioned medium obtained from 2-D A549 cultures did not modify tobramycin activity. Culturing epithelial cells of the lung or other mucosal tissues (such as the intestinal tract) as 3-D aggregates has been shown to alter phenotypic and biochemical properties, as compared to culture of these same cell types as 2-D monolayers [19,20,22,23,34,35]. In our study, 2-D A549 cells did not consume pyruvate nor produced the aminoglycoside-potentiating metabolite in the 2h time frame used to generate conditioned medium, in contrast to when these same cells were grown as 3-D structures. Succinate was produced at a concentration of 0.02 mM by 2-D cells, which was well below the threshold of 0.84 mM needed for tobramycin potentiation in this study. While the metabolome of cell cultures grown in a rotating wall vessel (RWV) bioreactor compared to as a monolayer has not been reported, several studies described metabolic differences in culturing human cells as spheroids versus monolayers [36,37]. For example, differences in glucose uptake, cellular proliferation, or differentiation might explain a different metabolism between 2-D and 3-D cultures that could have downstream effects on antibiotic potentiation [38,39]. Furthermore, in differentiated cells, oxidative phosphorylation in combination with the TCA cycle is the main pathway for the generation of ATP, while tumorigenic cells convert most glucose to lactate–referred to as the Warburg effect [40]. Hence, the reduced tumorigenic properties of 3-D A549 cells compared to 2-D cells [21] might be associated with a differential metabolism, favouring the use of the TCA cycle with the generation of potentiating metabolites as a result.

The aminoglycoside-potentiating activity of 3-D CM was confirmed for a broad range of *P*. *aeruginosa* strains and other *Pseudomonas* species. The observation that only few other bacterial species showed enhanced susceptibility to aminoglycosides was somewhat unexpected, especially since *E*. *coli* was reported to become more susceptible to aminoglycosides with supplementation of several TCA metabolites [2,7]. Strain- and species specific differences in intracellular pH (basal or in response to 3-D CM), membrane permeability (in particular for H^+^), metabolism (rate and type), and compensatory mechanisms might be responsible for this, and will be the subject of further study. In addition, while we chose antibiotic concentrations that resulted in at least 1 log biofilm inhibition in control conditions for all tested strains, concentration-dependent effects might also explain the observed differences in potentiation activity of 3-D CM between strains. Finally, we investigated possible alternative explanations for the potentiation of aminoglycosides by 3-D CM in this study, including the role of host-produced antimicrobial peptides (such as defensins). Overall, the results do not support a major involvement of antimicrobial peptides in the observations, as we would expect pronounced effects on membrane depolarization and permeability. The small increase in membrane permeability observed in the presence of tobramycin and 3-D CM is most likely attributable to the higher antibiotic uptake and activity, which indirectly leads to the generation of aberrant polypeptides that damage the cell membrane [41]. It should be mentioned that while host metabolites potentiated aminoglycoside activity under the experimental test conditions of this study (i.e. 4h biofilm inhibition assay, 24h time kill curve), other host-produced factors might influence activity of aminoglycosides and/or other antibiotics under different experimental conditions. For example, we observed a decrease in the MIC of colistin in 3-D CM compared to control (S1 Table), which is unlikely due to increased PMF; future studies will be required to elucidate the mechanism behind this potentiation.

In conclusion, our findings highlight the importance of tissue homeostasis in the innate defence against pathogens, through synergism between host metabolites and antibiotics. Our results support recent findings that the microenvironment of the host is a key player in determining antibiotic activity, and is important to consider when attempting to correlate antibiotic activity *in vitro* and *in vivo*. Our results also implicate that changes in metabolic activity of host cells, such as observed in lung epithelial cells of patients with cystic fibrosis [42], may impact the potentiating activity of lung epithelial cells towards aminoglycosides. Hence, supplementation of specific metabolites and/or *in vivo* stimulation of host metabolism might be a relevant approach to treat or prevent bacterial infections.

## Materials and methods

### Bacterial strains, growth media and conditions

An overview of all strains used in this study is presented in **Table 2**. All bacteria were cultured in Luria Bertani (LB) broth at 250 rpm and 37°C, with the exception of *P*. *fluorescens* which was cultured at 30°C and *Gemella haemolysans* which was grown in BHI broth. Bacteria were cultured until stationary phase for all experiments. When indicated, *P*. *aeruginosa* PAO1 and AA2 were cultured in M9 minimal medium supplemented with 10 mM glucose, 15 mM succinate or 12 mM glutamate (total carbon content of 60 mM). All bacteria were grown aerobically, except for *Streptococcus anginosus* and *G*. *haemolysans* which were grown under low oxygen conditions (±5% O_2_, ±15% CO_2_) using the CampyGen Compact system (Oxoid, Thermo Fisher Scientific).

**Table 2:** Strains used in this study.

Species	Strain number
**Reference strain**	
***P*. *aeruginosa***	PAO1 (ATCC 15692)
**Sputum isolates from cystic fibrosis patients**	
*P*. *aeruginosa*	AA44 ^[43]^
*P*. *aeruginosa*	AA2 ^[43]^
*P*. *aeruginosa*	AA43 ^[43]^
*P*. *aeruginosa*	LESB58 ^[43]^
*P*. *aeruginosa*	LES400 ^[43]^
*P*. *aeruginosa*	DK2 ^[43]^
*P*. *aeruginosa*	1709–12 ^[43]^
*P*. *aeruginosa*	Mi126 ^[43]^
*P*. *aeruginosa*	AMT0023-34 ^[43]^
**Environmental isolates**	
*P*. *aeruginosa*	Jpn1563 ^[43]^
*P*. *aeruginosa*	Pr335 ^[43]^
**Other *Pseudomonas* species**	
*P*. *putida*	KT2440 (ATCC 47054)
*P*. *fluorescens*	ATCC 17400
*P*. *stutzeri*	LMG 1228
**Other bacterial species**	
*Staphylococcus aureus*	SP123 ^[44]^
*Enterococcus facium*	LMG 16164
*Salmonella typhimurium*	ATCC 700720
*Acinetobacter baumannii*	AB5075 (ATCC 19606)
*Achromobacter xylosoxidans*	LMG 14980
*Burkholderia cenocepacia*	K56-2 (LMG 18863)
*Escherichia coli*	ATCC 25922
*Streptococcus anginosus*	LMG 14696
*Rothia mucilaginosa*	DSM 20746
*Gemella haemolysans*	LMG 18984
***P*. *aeruginosa* efflux pump mutants and isogenic wild type**	
*P*. *aeruginosa*	PAO1 ^[45]^
*P*. *aeruginosa*	PAO1 ΔmexXY ^[45]^
*P*. *aeruginosa*	PAO1 Δ MexAB::FRT ^[46]^

### Three-dimensional lung epithelial cell culture

An organotypic 3-D lung epithelial model was generated by culturing the human adenocarcinoma alveolar epithelial cell line A549 (ATCC CCL-185) on porous microcarrier beads in the RWV as described previously [19,21]. A549 cells were cultured in GTSF-2 medium (GE Healthcare) supplemented with 2.5 mg/L insulin transferrin selenite (ITS) (Sigma-Aldrich), 1.5 g/L sodium bicarbonate, and 10% heat-inactivated FBS (Invitrogen). All cultures were grown at 37°C under 5% CO_2_ and >80% humidity conditions. 3-D lung epithelial cultures were used for generation of 3-D conditioned medium after 11 to 14 days of growth in the RWV. The cell concentration in the RWV bioreactor was evaluated by treating an aliquot of the 3-D culture with 0.25% trypsin-EDTA (Life Technologies), followed by staining with 0.4% trypan blue (Sigma-Aldrich) and counting in a haemocytometer. Next, 2.5 x 10^5^ or 1 x 10^6^ viable cells/well were transferred in 48-well plates in a total volume of 250 μL fresh medium (corresponding with 10^6^ or 4 x 10^6^ cells/mL) for subsequent generation of 3-D CM.

### Two-dimensional lung epithelial cell culture

A549 cells were grown in T75 tissue culture flasks in GTSF-2 medium until reaching >70% confluence. The confluent monolayer was trypsinized with 0.25% trypsin-EDTA (Life Technologies) and 10^5^ viable cells/well were transferred into 6-well plates and allowed to reach confluence (2–3 days).

### 3-D and 2-D conditioned medium

3-D A549 lung epithelial cells distributed in 48-well plates at a concentration of 2.5 x 10^5^ or 1 x 10^6^ cells/well (in 250 μL fresh GTSF-2 medium/well) were incubated for 2h at 37°C, 5% CO2, >80% humidity. Then, the cell culture medium was collected and filtered through a 0.22 μm low protein binding filter (Millipore) to remove cell debris, resulting in 3-D conditioned medium (3-D CM). A549 cells grown to confluence as monolayers in a 6-well plate were trypsinized to determine the total cell number per well, and fresh cell culture medium was added to intact monolayers to obtain a concentration of 1 x 10^6^ cells/mL. After 2h incubation, conditioned medium of 2-D A549 cells (2-D CM) was obtained using the same protocol as for 3-D CM.

For experiments aiming to determine the role of bicarbonate in the potentiating effect of 3-D CM, GTSF-2 that was not supplemented with 1.5 g/L bicarbonate was used to generate 3-D CM. When indicated, 3-D CM was filtered over a 3 kDa filter (Millipore), or treated with 2 μg/mL trypsin (Promega) or 476 μg/mL proteinase K (Sigma) following the manufacturer’s instructions.

### Biofilm inhibition assay

Inhibition of biofilm formation by antibiotics in 3-D CM, 2-D CM or control medium (GTSF-2) on a plastic surface was determined as described previously [47], with modifications. Where indicated, biofilm inhibition was evaluated in control medium supplemented with glutamate (final concentration 10.88 mM) and/or succinate (final concentration 1.81 mM or concentration range) or CM/control media were supplemented with triphenylbismuthdichloride (16, 64, 128 μg/mL), CCCP (100 μM) or KCl (150 mM) and nigericin (10 μM) (Sigma-Aldrich). Briefly, bacterial cultures grown to stationary phase were diluted to an OD of 0.05 and transferred to 96-well plates. Antimicrobial agents were added at a concentration that inhibited biofilm formation by at least one log unit in the control medium, i.e. for *P*. *aeruginosa* PAO1 2 μg/mL for tobramycin (Sigma-Aldrich), 4 μg/mL for amikacin (TCI), 8 μg/mL for gentamicin (Sigma-Aldrich) and 2 μg/mL for colistin (TCI). Antibiotic concentrations for other *P*. *aeruginosa* strains, *Pseudomonas* species and other bacterial species were also chosen to obtain biofilm inhibition of at least one log unit in control medium and are listed in Table 1. Plates were incubated for 4 h in a 37°C, 5% CO_2_ incubator, and the number of colony forming units (CFU) attached to the surface was determined by homogenizing the biofilm through two rounds of vortexing (900 rpm, 5 min) and sonication (5 min; Branson Ultrasonic bath). The homogenized biofilms were serially diluted and plated on tryptic soy agar (TSA) for all bacteria (detection limit = 10^2^ CFU/mL), except *G*. *haemolysans* which was plated on Columbia agar with 6% sheep blood. Plates were incubated at 37°C overnight (16 h) or until colonies could be counted.

### Biofilm eradication assay

Biofilm eradication in 3-D CM or control medium (GTSF-2) by antibiotics was performed as described previously [48]. Stationary phase cultures were diluted to an OD of 0.05, transferred to 96-well plates and incubated at 37°C for 24h to allow biofilm formation. Then, biofilms were rinsed and subsequently treated with antibiotics dissolved in the 3-D CM or control medium. Antibiotic concentrations were chosen to obtain at least 1 log biofilm eradication in the control medium, i.e. 15 μg/mL for tobramycin (Sigma-Aldrich), 35 and 50 μg/mL for amikacin (TCI), 25 μg/mL for gentamicin (Sigma-Aldrich) and 500 μg/mL for colistin (TCI). Biofilms were homogenized and CFU were determined as described for the biofilm inhibition assay.

### Minimal inhibitory concentration

The minimal inhibitory concentration (MIC) was determined according to EUCAST guidelines. When indicated, the MIC was determined in control cell culture medium (GTSF-2) or 3-D CM instead of Mueller Hinton (MH) Broth.

### Time-kill curve

Stationary phase cultures were diluted to an OD of 0.05 in 3-D CM or control medium, and tobramycin was added at concentrations 2-fold or 8-fold higher than the MIC determined according to the EUCAST guidelines. Cultures were incubated in a 37°C shaking incubator (250 rpm) up to 24h. At the start of the experiment and at every indicated time point, an aliquot of the culture was serially diluted and plated on TSA or LB agar to determine the CFU/mL (detection limit = 10^2^ CFU/mL).

### Intracellular tobramycin levels

BODIPY-labelled tobramycin (**S7 Fig**) was synthesized according to the protocol provided in the Supporting Information (**S1 Text**). The biofilm formation assay was performed in the presence of varying levels of BODIPY-tobramycin (as indicated) in 3-D CM or control medium at 37°C, or at 4°C when indicated. Following 4h of biofilm formation, the biofilm was rinsed to remove extracellular tobramycin, homogenized and subjected to flow cytometry analysis (Attune NxT, Life Technologies). The bacterial population was delineated based on the forward and side scatter signal, and a threshold was set to exclude non-cellular particles and cell debris. BODIPY-tobramycin that associated with bacterial cells was determined through excitation with a 488 nm laser. Fluorescence emission was detected through a 530/30 bandpass filter. Controls included bacterial biofilm cells that were not exposed to BODIPY-tobramycin (negative control) or to incremental levels of tobramycin to determine the concentration at which saturation was obtained. Based on the negative control and the concentration of tobramycin where maximal population saturation was obtained, negative and positive flow cytometry gates were determined respectively. The intermediate gate contained bacterial cells located in between the negative and positive gates (**S2 Fig**). At least 10,000 bacteria were analysed per sample.

### Intracellular pH

Intracellular pH was measured by a 2,7 -Bis(2-carboxyethyl)-5(6)-carboxyfluorescein acetoxymethyl (BCECF-AM) assay as described previously [5], with modifications. Briefly, stationary phase cultures were exposed to 25 μM BCECF-AM (Sigma-Aldrich) for 30 min at 30°C. Loaded cells were washed twice with 0.9% NaCl and resuspended in fresh LB medium. Next, the same protocol as described for biofilm inhibition in 3-D CM versus control medium (in the presence and absence of 2 μg/mL tobramycin) was used with BCECF-AM-loaded cultures. Fluorescence was measured every 20 s for 30 min as a ratio of emission at 535 nm with dual wavelength excitation at 480 nm and 450 nm, using a plate-reader spectrophotometer (Envision, Perkin Elmer). Each experiment included an intracellular pH calibration curve using control medium at a pH range of 6 to 8. To this end, bacteria were exposed to GTSF-2 medium supplemented with excess KCl (150 mM) and nigericin (10 μM) to equilibrate intracellular and extracellular pH.

### Membrane depolarization and permeability

A fluorimetric assay was used to measure cell membrane depolarization of *P*. *aeruginosa*, using the membrane potential-sensitive dye DiBAC_4_(3) (Bis-(1,3-Dibarbituric acid)-trimethine oxanol), as described previously with modifications [49]. Briefly, stationary phase cultures were diluted fifty times in fresh LB medium, and cultured until early logarithmic phase (OD ~0.5). Cultures were pelleted and resuspended in control medium or 3-D CM, and antibiotics were added at the indicated concentration. After 4h incubation, samples were taken and exposed to a final concentration of 10 μg/mL DiBAC_4_(3) (from a 10 mg/mL stock in DMSO) (Invitrogen) for 5 min at 37°C in the dark. Bacteria were pelleted, resuspended in filtered 0.9% NaCl, and diluted 100x in filtered 0.9% NaCl. Samples were allowed to equilibrate for 15 min at room temperature prior to flow cytometry analysis. Fluorescence emission due to membrane depolarization was measured using a flow cytometer (Attune NXT, Thermofisher) equipped with a 530/30 bandpass filter and 488 nm light source. At least 10,000 bacteria were analyzed, which were delineated based on the forward scatter (FSC) and side scatter (SSC) signal. A threshold on the FSC and SSC was set to exclude debris and non-cellular particles. To evaluate membrane permeability, a LIVE/DEAD BacLight Bacterial Viability Kit was used (Thermofisher), and manufacturer’s instructions were followed. Live, dead, and intermediate populations were determined based on the fluorescence emission detected with 530/30 and 695/40 bandpass filters, following excitation at 488 nm, as described previously [30].

### Quantification of pyruvate, glutamate and succinate

Host metabolites were quantified using colorimetric or fluorometric assay kits for pyruvate, succinate, glutamate (Abcam), according to the manufacturer’s protocols.

### Statistical analysis

All experiments were performed at least in biological triplicate, and with 1–3 technical replicates. For the graphs where standard error mean was used to present variability, the number of replicates per data point is provided in **S2 Table**. Statistical analysis of quantitative assays was done using SPSS statistics software version 25. The Shapiro–Wilk test was used in combination with Q/Q plot analysis to verify the normal distribution of the data. For normally distributed data, assessment of equality of variances was performed using a Levene’s test, followed by an independent sample t-test. Data sets that were not normally distributed were analysed using a Mann–Whitney test. For data sets involving multiple sample comparisons of normally distributed data, ANOVA-testing was performed followed by a Dunnett’s post hoc analysis. When normality was not confirmed, a Kruskal-Wallis non-parametric test was done. P-values <0.05 were considered statistically significant.

## Supporting information

S1 Fig Antibiotic activity against *P*. *aeruginosa* PAO1 established biofilms in conditioned medium derived from 3-D lung epithelial cells (4 x 10^6^ cells/mL). Biofilms were formed for 24h in control medium (GTSF-2), and were treated subsequently for an additional 24h with antibiotics in the presence of 3-D CM or control medium. Col = colistin, Am = amikacin, Tb = tobramycin, Gm = gentamicin. No significant differences were observed between control and 3-D CM. Error bars represent standard error of the mean.
(TIF)

S2 Fig Determination of flow cytometry gates to evaluate bacterial uptake of BODIPY-tobramycin. An untreated sample was used to delineate the “negative gate” (Neg). To determine the “positive gate” (Pos), fluorescence intensity was determined using increasing concentrations of BODIPY-tobramycin, until the maximal fluorescence intensity was reached (i.e. no further increase with higher concentrations). The positive gate contoured the bacterial population with maximal fluorescence intensity. Bacteria that were situated in between the negative and positive gates, were captured in the “intermediate gate” (Int). Dot plots present the forward scatter signal intensity in the X-axis, and the BODIPY-tobramycin fluorescence intensity in the Y-axis.
(TIF)

S3 Fig Uptake of BODIPY-tobramycin by *P*. *aeruginosa* PAO1 (A) and DK2 (B) in 3-D CM versus control medium, determined using flow cytometry analysis. Tobramycin uptake was assessed based on the fraction of the population that fell into three respective gates: negative, intermediate and positive. Negative and positive gates were determined respectively using a negative control (untreated sample) and BODIPY-tobramycin concentrations that resulted in maximal fluorescence intensity (S2 Fig). Bacteria whose fluorescence was situated between the positive and negative gates represent the intermediate population. Biofilms were formed for 4h in the presence of 0.5 μg/mL BODIPY-tobramycin. * p < 0.05, ** p < 0.01, n ≥ 3. Error bars represent standard error of the mean.
(TIF)

S4 Fig Uptake of BODIPY-tobramycin (0.75 μg/mL) by *P*. *aeruginosa* PAO1 in 3-D CM and control medium for 4h at 4°C. Panel A is derived from the dot plot graphs (left and middle image) in panel B (forward scatter signal in X-axis, fluorescence intensity in Y-axis) (representative replicate). The right image of panel B presents an overlay of the histograms from control and 3-D CM samples, showing the fluorescence intensity on the X-axis and the percentage of the analysed cell population in the Y-axis. Control medium was GTSF-2. * p < 0.05, n ≥ 3. Error bars represent standard error of the mean.
(TIF)

S5 Fig Influence of pH of 3-D CM on tobramycin potentiation. Biofilm inhibition (4h) of *P*. *aeruginosa* PAO1 in the presence of control medium, or 3-D CM maintained at its original pH (pH 6.99) or adjusted to the pH of control medium (pH 7.29). Control medium is GTSF-2. ** p < 0.01, n ≥ 3. Error bars represent standard error of the mean.
(TIF)

S6 Fig Role of host-produced peptides and bicarbonate in the potentiation effect of 3-D CM. (A) Biofilm inhibition by 2 μg/mL tobramycin in control medium and 3-D CM filtered (3 kDa) treated with proteinase K or solvent control. (B) Biofilm inhibition of *P*. *aeruginosa* PAO1 by 2 μg/mL tobramycin in control medium and 3-D CM filtered treated with trypsin or solvent control. (C) Biofilm inhibition by 2 μg/mL tobramycin in 3-D CM or control medium containing 1.5 g/L bicarbonate or no bicarbonate. Control medium was GTSF-2. ** p < 0.01, n ≥ 3. Error bars represent standard error of the mean.
(TIF)

S7 Fig Structure of BODIPY-labelled tobramycin. (TIF)

S1 Table MIC90 of *P*. *aeruginosa* in control medium (GTSF-2) or 3-D CM. (TIF)

S2 Table Number of replicates per data point for which standard error mean is presented. (XLSX)

S1 Text Synthesis of BODIPY-tobramycin. Schematic overview of the BODIPY-tobramycin synthesis process and NMR spectra for synthesized BODIPY-tobramycin.
(PDF)

## References

[1] Peng B, Su YB, Li H, Han Y, Guo C, Tian YM, et al (2015) Exogenous alanine and/or glucose plus kanamycin kills antibiotic-resistant bacteria. Cell Metab 21: 249–262. 10.1016/j.cmet.2015.01.008 25651179

[2] Allison KR, Brynildsen MP, Collins JJ (2011) Metabolite-enabled eradication of bacterial persisters by aminoglycosides. Nature 473: 216–220. 10.1038/nature10069 21562562 PMC3145328

[3] Yang JH, Bening SC, Collins JJ (2017) Antibiotic efficacy-context matters. Curr Opin Microbiol 39: 73–80. 10.1016/j.mib.2017.09.002 29049930 PMC5732053

[4] Yang JH, Bhargava P, McCloskey D, Mao N, Palsson BO, Collins JJ (2017) Antibiotic-Induced Changes to the Host Metabolic Environment Inhibit Drug Efficacy and Alter Immune Function. Cell Host Microbe 22: 757–765 e753. 10.1016/j.chom.2017.10.020 29199098 PMC5730482

[5] Farha MA, French S, Stokes JM, Brown ED (2018) Bicarbonate Alters Bacterial Susceptibility to Antibiotics by Targeting the Proton Motive Force. ACS Infect Dis 4: 382–390. 10.1021/acsinfecdis.7b00194 29264917

[6] Su YB, Peng B, Li H, Cheng ZX, Zhang TT, Zhu JX, et al (2018) Pyruvate cycle increases aminoglycoside efficacy and provides respiratory energy in bacteria. Proc Natl Acad Sci U S A 115: E1578–E1587. 10.1073/pnas.1714645115 29382755 PMC5816162

[7] Meylan S, Porter CBM, Yang JH, Belenky P, Gutierrez A, Lobritz MA, et al (2017) Carbon Sources Tune Antibiotic Susceptibility in Pseudomonas aeruginosa via Tricarboxylic Acid Cycle Control. Cell Chem Biol 24: 195–206. 10.1016/j.chembiol.2016.12.015 28111098 PMC5426816

[8] Su YB, Peng B, Han Y, Li H, Peng XX (2015) Fructose restores susceptibility of multidrug-resistant Edwardsiella tarda to kanamycin. J Proteome Res 14: 1612–1620. 10.1021/pr501285f 25675328

[9] Ersoy SC, Heithoff DM, Barnes Lt, Tripp GK, House JK, Marth JD, et al (2017) Correcting a Fundamental Flaw in the Paradigm for Antimicrobial Susceptibility Testing. EBioMedicine 20: 173–181. 10.1016/j.ebiom.2017.05.026 28579300 PMC5478264

[10] Lebeaux D, Chauhan A, Letoffe S, Fischer F, de Reuse H, Beloin C, et al (2014) pH-mediated potentiation of aminoglycosides kills bacterial persisters and eradicates in vivo biofilms. J Infect Dis 210: 1357–1366. 10.1093/infdis/jiu286 24837402

[11] Hurley MN, Ariff AH, Bertenshaw C, Bhatt J, Smyth AR (2012) Results of antibiotic susceptibility testing do not influence clinical outcome in children with cystic fibrosis. J Cyst Fibros 11: 288–292. 10.1016/j.jcf.2012.02.006 22436723 PMC3382712

[12] Thulin E, Sundqvist M, Andersson DI (2015) Amdinocillin (Mecillinam) resistance mutations in clinical isolates and laboratory-selected mutants of *Escherichia* coli. Antimicrobial agents and chemotherapy 59: 1718–1727. 10.1128/AAC.04819-14 25583718 PMC4325821

[13] McKinnell J, Classi P, Blumberg P, Murty S, Tillotson G. Clinical predictors of antibiotic failure in adult outpatients with community-acquired pneumonia; 2017 195:A2644; Washington DC, USA.

[14] Hughes D, Andersson DI (2017) Environmental and genetic modulation of the phenotypic expression of antibiotic resistance. FEMS microbiology reviews 41: 374–391. 10.1093/femsre/fux004 28333270 PMC5435765

[15] Pan X, Dong Y, Fan Z, Liu C, Xia B, Shi J, et al (2017) In vivo Host Environment Alters *Pseudomonas aeruginosa* Susceptibility to Aminoglycoside Antibiotics. Front Cell Infect Microbiol 7: 83 10.3389/fcimb.2017.00083 28352614 PMC5348532

[16] Anderson GG, Moreau-Marquis S, Stanton BA, O'Toole GA (2008) In vitro analysis of tobramycin-treated *Pseudomonas aeruginosa* biofilms on cystic fibrosis-derived airway epithelial cells. Infect Immun 76: 1423–1433. 10.1128/IAI.01373-07 18212077 PMC2292855

[17] Moreau-Marquis S, Bomberger JM, Anderson GG, Swiatecka-Urban A, Ye S, O'Toole GA, et al (2008) The DeltaF508-CFTR mutation results in increased biofilm formation by *Pseudomonas aeruginosa* by increasing iron availability. Am J Physiol Lung Cell Mol Physiol 295: L25–37. 10.1152/ajplung.00391.2007 18359885 PMC2494796

[18] Moreau-Marquis S, Stanton BA, O'Toole GA (2008) *Pseudomonas aeruginosa* biofilm formation in the cystic fibrosis airway. Pulm Pharmacol Ther 21: 595–599. 10.1016/j.pupt.2007.12.001 18234534 PMC2542406

[19] Crabbe A, Liu Y, Matthijs N, Rigole P, De La Fuente-Nunez C, Davis R, et al (2017) Antimicrobial efficacy against *Pseudomonas aeruginosa* biofilm formation in a three-dimensional lung epithelial model and the influence of fetal bovine serum. Sci Rep 7: 43321 10.1038/srep43321 28256611 PMC5335707

[20] Barrila J, Radtke AL, Crabbe A, Sarker SF, Herbst-Kralovetz MM, Ott CM, et al (2010) Organotypic 3D cell culture models: using the rotating wall vessel to study host-pathogen interactions. Nat Rev Microbiol 8: 791–801. 10.1038/nrmicro2423 20948552

[21] Carterson AJ, Honer zu Bentrup K, Ott CM, Clarke MS, Pierson DL, Vanderburg CR, et al (2005) A549 lung epithelial cells grown as three-dimensional aggregates: alternative tissue culture model for *Pseudomonas aeruginosa* pathogenesis. Infect Immun 73: 1129–1140. 10.1128/IAI.73.2.1129-1140.2005 15664956 PMC547019

[22] Crabbé A, Ledesma MA, Nickerson CA (2014) Mimicking the host and its microenvironment in vitro for studying mucosal infections by *Pseudomonas aeruginosa*. Pathog Dis 71: 1–19. 10.1111/2049-632X.12180 24737619 PMC4096993

[23] Crabbé A, Sarker SF, Van Houdt R, Ott CM, Leys N, Cornelis P, et al (2011) Alveolar epithelium protects macrophages from quorum sensing-induced cytotoxicity in a three-dimensional co-culture model. Cellular Microbiology 13: 469–481. 10.1111/j.1462-5822.2010.01548.x 21054742

[24] Taber HW, Mueller JP, Miller PF, Arrow AS (1987) Bacterial uptake of aminoglycoside antibiotics. Microbiol Rev 51: 439–457. 3325794 10.1128/mr.51.4.439-457.1987 PMC373126

[25] Hancock RE, Bell A (1988) Antibiotic uptake into gram-negative bacteria. Eur J Clin Microbiol Infect Dis 7: 713–720. 2850910 10.1007/BF01975036

[26] Chopra I (1988) Molecular mechanisms involved in the transport of antibiotics into bacteria. Parasitology 96 Suppl: S25–44. 3287290 10.1017/s0031182000085966

[27] Kasianowicz J, Benz R, McLaughlin S (1984) The kinetic mechanism by which CCCP (carbonyl cyanide m-chlorophenylhydrazone) transports protons across membranes. J Membr Biol 82: 179–190. 6096547 10.1007/BF01868942

[28] Li XZ, Poole K, Nikaido H (2003) Contributions of MexAB-OprM and an EmrE homolog to intrinsic resistance of *Pseudomonas aeruginosa* to aminoglycosides and dyes. Antimicrob Agents Chemother 47: 27–33. 10.1128/AAC.47.1.27-33.2003 12499164 PMC149025

[29] Masuda N, Sakagawa E, Ohya S, Gotoh N, Tsujimoto H, Nishino T (2000) Contribution of the MexX-MexY-oprM efflux system to intrinsic resistance in *Pseudomonas aeruginosa*. Antimicrob Agents Chemother 44: 2242–2246. 10952562 10.1128/aac.44.9.2242-2246.2000 PMC90052

[30] Berney M, Hammes F, Bosshard F, Weilenmann HU, Egli T (2007) Assessment and interpretation of bacterial viability by using the LIVE/DEAD BacLight Kit in combination with flow cytometry. Appl Environ Microbiol 73: 3283–3290. 10.1128/AEM.02750-06 17384309 PMC1907116

[31] Birkenstock T, Liebeke M, Winstel V, Krismer B, Gekeler C, Niemiec MJ, et al (2012) Exometabolome analysis identifies pyruvate dehydrogenase as a target for the antibiotic triphenylbismuthdichloride in multiresistant bacterial pathogens. The Journal of biological chemistry 287: 2887–2895. 10.1074/jbc.M111.288894 22144679 PMC3268445

[32] Hancock RE, Scott MG (2000) The role of antimicrobial peptides in animal defenses. Proc Natl Acad Sci U S A 97: 8856–8861. 10922046 10.1073/pnas.97.16.8856 PMC34023

[33] Slachmuylders L, Van Acker H, Brackman G, Sass A, Van Nieuwerburgh F, Coenye T (2018) Elucidation of the mechanism behind the potentiating activity of baicalin against *Burkholderia cenocepacia* biofilms. PloS one 13: e0190533 10.1371/journal.pone.0190533 29293658 PMC5749847

[34] Barrila J, Yang J, Crabbé A, Sarker SF, Liu Y, Ott CM, et al (2017) Three-dimensional organotypic co-culture model of intestinal epithelial cells and macrophages to study *Salmonella enterica* colonization patterns. NPJ Microgravity 3: 10 10.1038/s41526-017-0011-2 28649632 PMC5460263

[35] Barrila J, Crabbe A, Yang J, Franco K, Nydam SD, Forsyth RJ, et al (2018) Modeling Host-Pathogen Interactions in the Context of the Microenvironment: Three-Dimensional Cell Culture Comes of Age. Infection and immunity 86. 10.1128/IAI.00282-18 PMC6204695 30181350

[36] Russell S, Wojtkowiak J, Neilson A, Gillies RJ (2017) Metabolic Profiling of healthy and cancerous tissues in 2D and 3D. Sci Rep 7: 15285 10.1038/s41598-017-15325-5 29127321 PMC5681543

[37] Prina-Mello A, Jain N, Liu B, Kilpatrick JI, Tutty MA, Bell AP, et al (2018) Culturing substrates influence the morphological, mechanical and biochemical features of lung adenocarcinoma cells cultured in 2D or 3D. Tissue Cell 50: 15–30. 10.1016/j.tice.2017.11.003 29429514

[38] Chitcholtan K, Asselin E, Parent S, Sykes PH, Evans JJ (2013) Differences in growth properties of endometrial cancer in three dimensional (3D) culture and 2D cell monolayer. Exp Cell Res 319: 75–87. 10.1016/j.yexcr.2012.09.012 23022396

[39] Duval K, Grover H, Han LH, Mou Y, Pegoraro AF, Fredberg J, et al (2017) Modeling Physiological Events in 2D vs. 3D Cell Culture. Physiology (Bethesda) 32: 266–277. 28615311 10.1152/physiol.00036.2016 PMC5545611

[40] Eisenreich W, Heesemann J, Rudel T, Goebel W (2013) Metabolic host responses to infection by intracellular bacterial pathogens. Frontiers in cellular and infection microbiology 3: 24 10.3389/fcimb.2013.00024 23847769 PMC3705551

[41] Davis BD, Chen LL, Tai PC (1986) Misread protein creates membrane channels: an essential step in the bactericidal action of aminoglycosides. Proceedings of the National Academy of Sciences of the United States of America 83: 6164–6168. 2426712 10.1073/pnas.83.16.6164 PMC386460

[42] Wetmore DR, Joseloff E, Pilewski J, Lee DP, Lawton KA, Mitchell MW, et al (2010) Metabolomic profiling reveals biochemical pathways and biomarkers associated with pathogenesis in cystic fibrosis cells. J Biol Chem 285: 30516–30522. 10.1074/jbc.M110.140806 20675369 PMC2945545

[43] Cullen L, Weiser R, Olszak T, Maldonado RF, Moreira AS, Slachmuylders L, et al (2015) Phenotypic characterization of an international Pseudomonas aeruginosa reference panel: strains of cystic fibrosis (CF) origin show less in vivo virulence than non-CF strains. Microbiology 161: 1961–1977. 10.1099/mic.0.000155 26253522

[44] Vandecandelaere I, Matthijs N, Nelis HJ, Depuydt P, Coenye T (2013) The presence of antibiotic-resistant nosocomial pathogens in endotracheal tube biofilms and corresponding surveillance cultures. Pathog Dis 69: 142–148. 10.1111/2049-632X.12100 24115610

[45] Robertson GT, Doyle TB, Du Q, Duncan L, Mdluli KE, Lynch AS (2007) A Novel indole compound that inhibits *Pseudomonas aeruginosa* growth by targeting MreB is a substrate for MexAB-OprM. J Bacteriol 189: 6870–6881. 10.1128/JB.00805-07 17644596 PMC2045200

[46] Mima T, Joshi S, Gomez-Escalada M, Schweizer HP (2007) Identification and characterization of TriABC-OpmH, a triclosan efflux pump of *Pseudomonas aeruginosa* requiring two membrane fusion proteins. J Bacteriol 189: 7600–7609. 10.1128/JB.00850-07 17720796 PMC2168734

[47] Brackman G, Cos P, Maes L, Nelis HJ, Coenye T (2011) Quorum sensing inhibitors increase the susceptibility of bacterial biofilms to antibiotics in vitro and in vivo. Antimicrob Agents Chemother 55: 2655–2661. 10.1128/AAC.00045-11 21422204 PMC3101409

[48] Vandeplassche E, Coenye T, Crabbe A (2017) Developing selective media for quantification of multispecies biofilms following antibiotic treatment. PLoS One 12: e0187540 10.1371/journal.pone.0187540 29121069 PMC5679531

[49] Krahn T, Gilmour C, Tilak J, Fraud S, Kerr N, Lau CH, et al (2012) Determinants of intrinsic aminoglycoside resistance in *Pseudomonas aeruginosa*. Antimicrob Agents Chemother 56: 5591–5602. 10.1128/AAC.01446-12 22908149 PMC3486610

