# Supplementary figures and images for: Host metabolites stimulate the bacterial proton motive force to enhance the activity of aminoglycoside antibiotics
Source: PLoS Pathog. 2019 Apr 29;15(4):e1007697. doi: 10.1371/journal.ppat.1007697 (PMC6508747; doi:10.1371/journal.ppat.1007697)

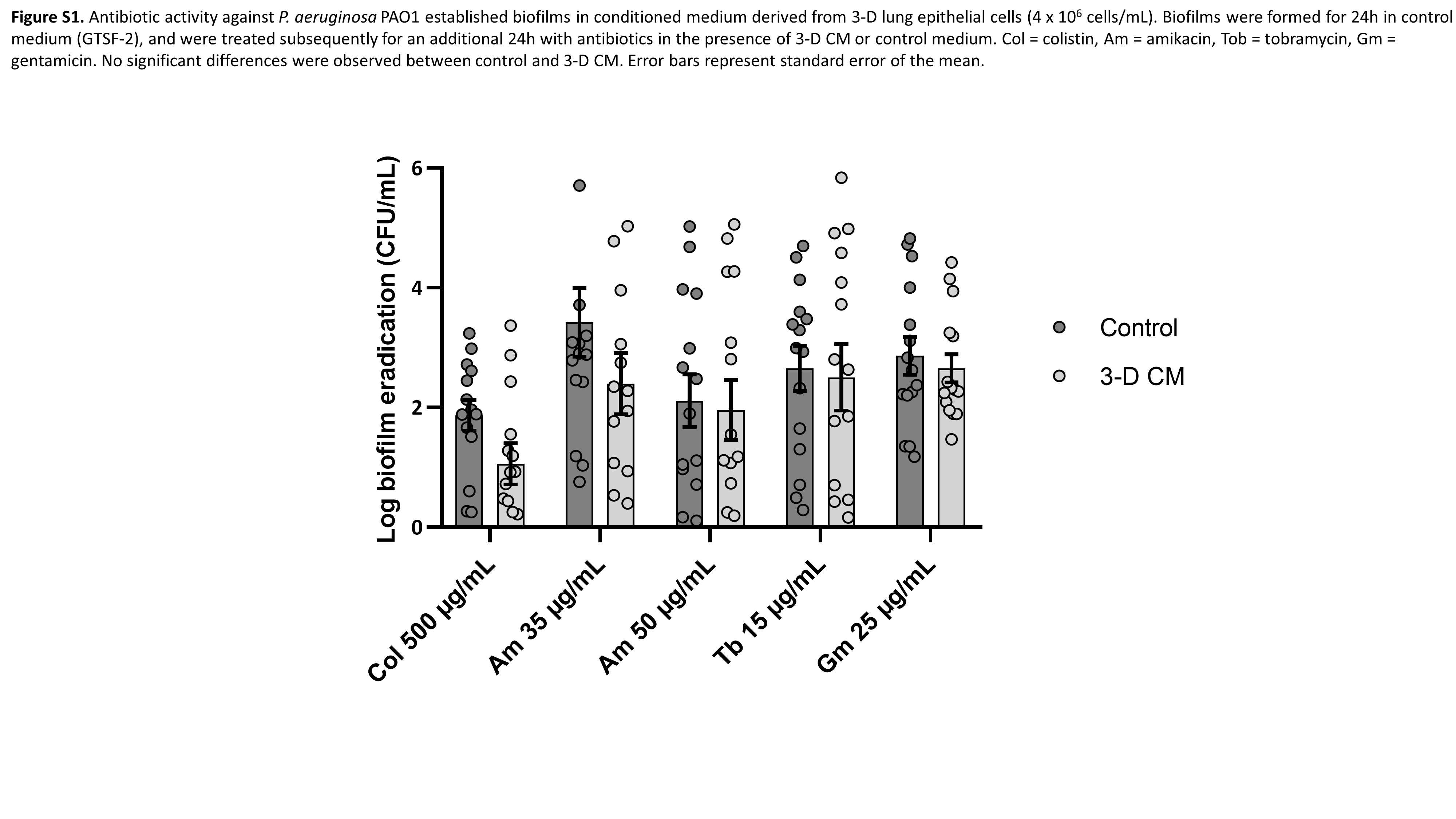

Supplement: S1 Fig — Biofilms were formed for 24h in control medium (GTSF-2), and were treated subsequently for an additional 24h with antibiotics in the presence of 3-D CM or control medium. Col = colistin, Am = amikacin, Tb = tobramycin, Gm = gentamicin. No significant differences were observed between control and 3-D CM. Error bars represent standard error of the mean. (TIF) [file ppat.1007697.s001.TIF]

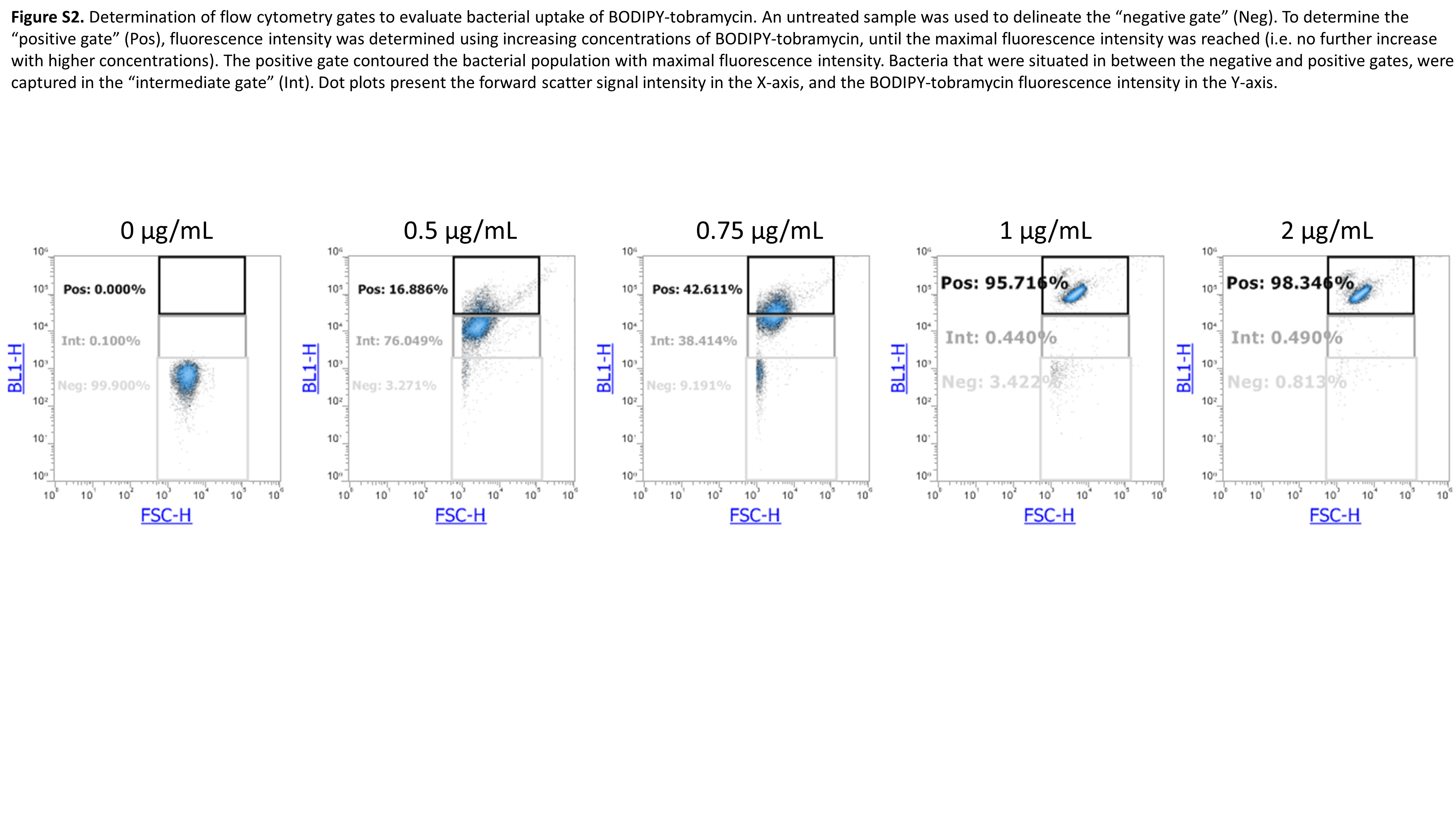

Supplement: S2 Fig — An untreated sample was used to delineate the “negative gate” (Neg). To determine the “positive gate” (Pos), fluorescence intensity was determined using increasing concentrations of BODIPY-tobramycin, until the maximal fluorescence intensity was reached (i.e. no further increase with higher concentrations). The positive gate contoured the bacterial population with maximal fluorescence intensity. Bacteria that were situated in between the negative and positive gates, were captured in the “intermediate gate” (Int). Dot plots present the forward scatter signal intensity in the X-axis, and the BODIPY-tobramycin fluorescence intensity in the Y-axis. (TIF) [file ppat.1007697.s002.TIF]

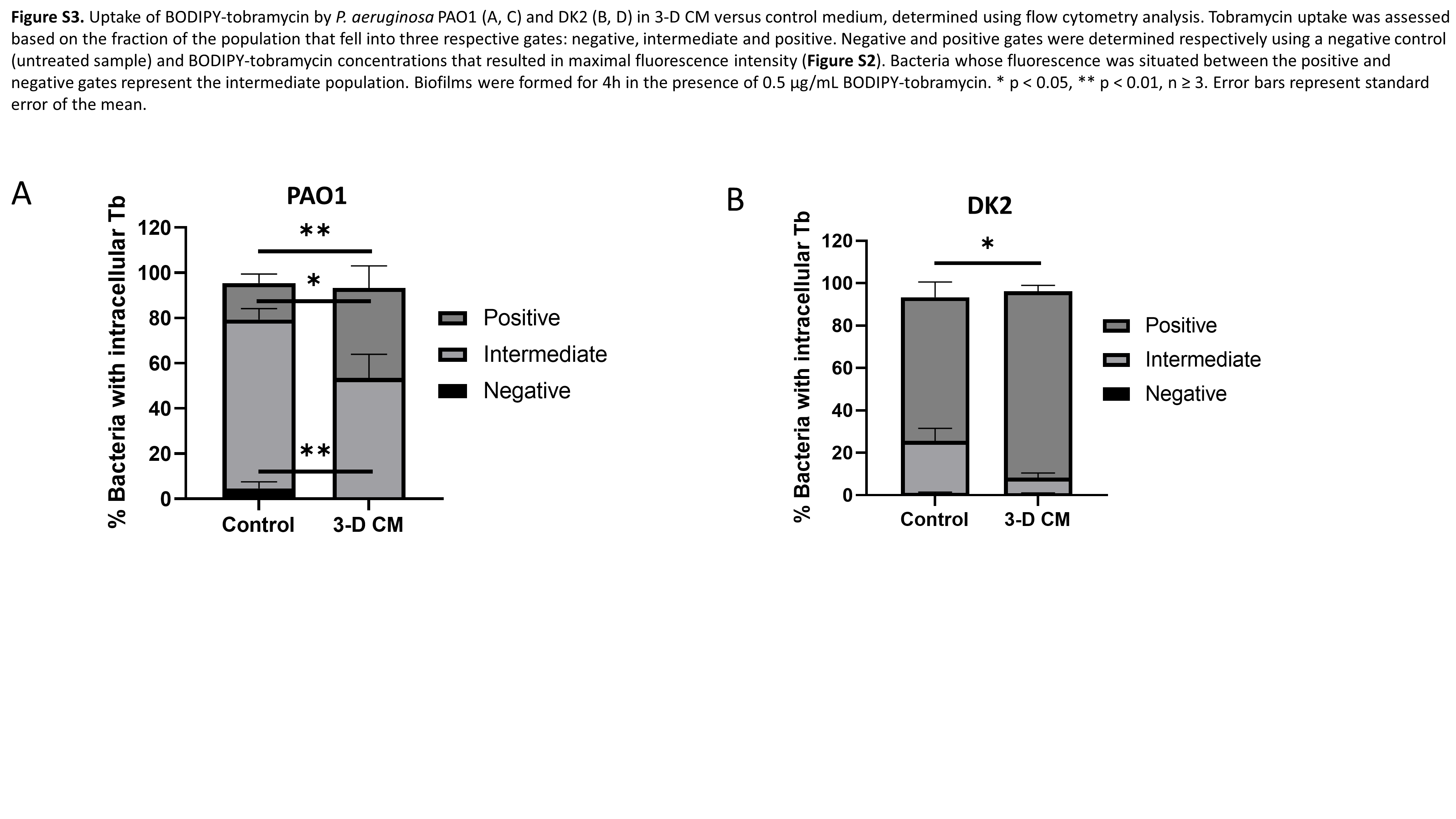

Supplement: S3 Fig — Uptake of BODIPY-tobramycin by P. aeruginosa PAO1 (A) and DK2 (B) in 3-D CM versus control medium, determined using flow cytometry analysis. Tobramycin uptake was assessed based on the fraction of the population that fell into three respective gates: negative, intermediate and positive. Negative and positive gates were determined respectively using a negative control (untreated sample) and BODIPY-tobramycin concentrations that resulted in maximal fluorescence intensity (S2 Fig). Bacteria whose fluorescence was situated between the positive and negative gates represent the intermediate population. Biofilms were formed for 4h in the presence of 0.5 μg/mL BODIPY-tobramycin. * p < 0.05, ** p < 0.01, n ≥ 3. Error bars represent standard error of the mean. (TIF) [file ppat.1007697.s003.TIF]

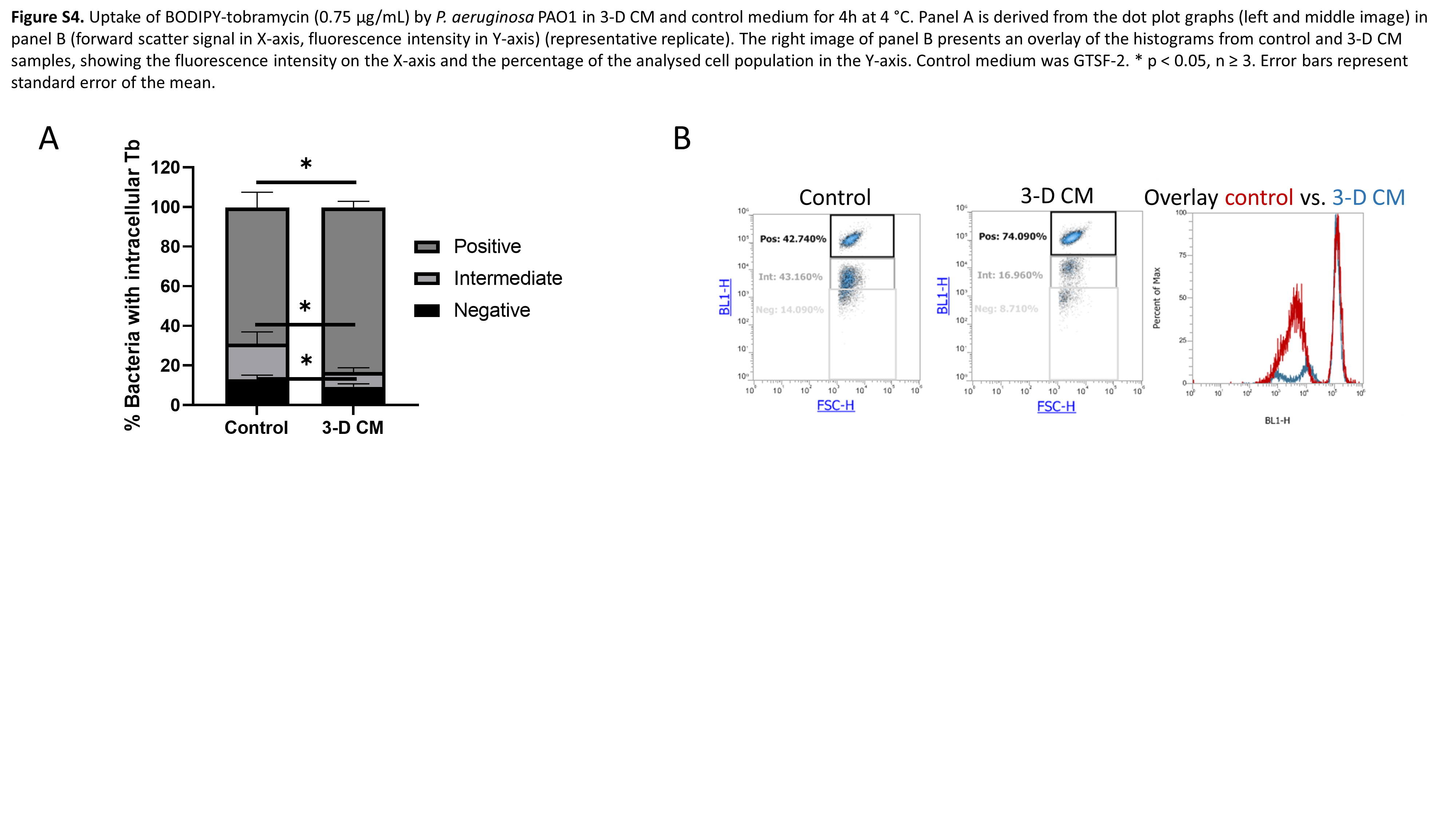

Supplement: S4 Fig — Panel A is derived from the dot plot graphs (left and middle image) in panel B (forward scatter signal in X-axis, fluorescence intensity in Y-axis) (representative replicate). The right image of panel B presents an overlay of the histograms from control and 3-D CM samples, showing the fluorescence intensity on the X-axis and the percentage of the analysed cell population in the Y-axis. Control medium was GTSF-2. * p < 0.05, n ≥ 3. Error bars represent standard error of the mean. (TIF) [file ppat.1007697.s004.TIF]

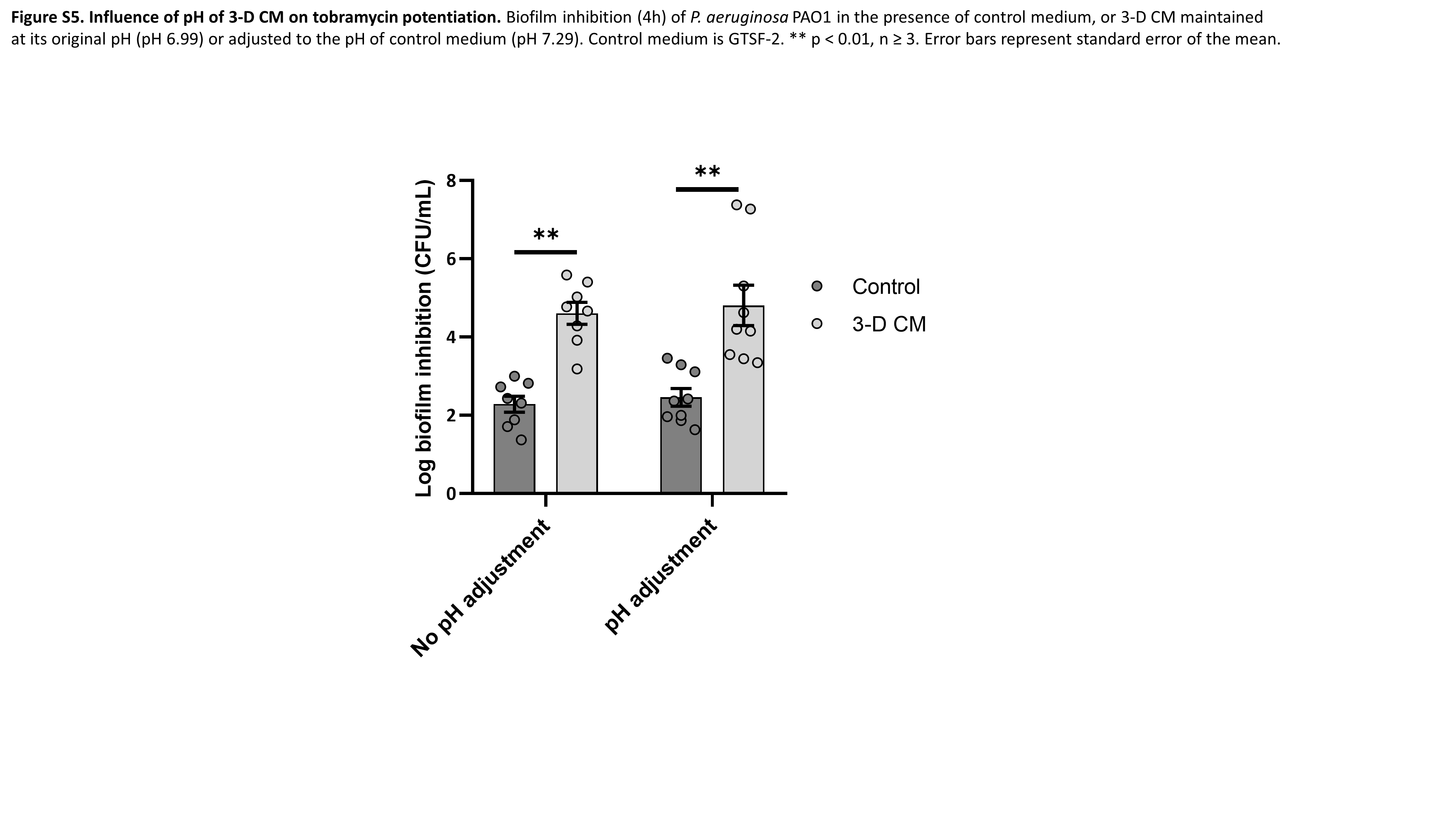

Supplement: S5 Fig — Biofilm inhibition (4h) of P. aeruginosa PAO1 in the presence of control medium, or 3-D CM maintained at its original pH (pH 6.99) or adjusted to the pH of control medium (pH 7.29). Control medium is GTSF-2. ** p < 0.01, n ≥ 3. Error bars represent standard error of the mean. (TIF) [file ppat.1007697.s005.TIF]

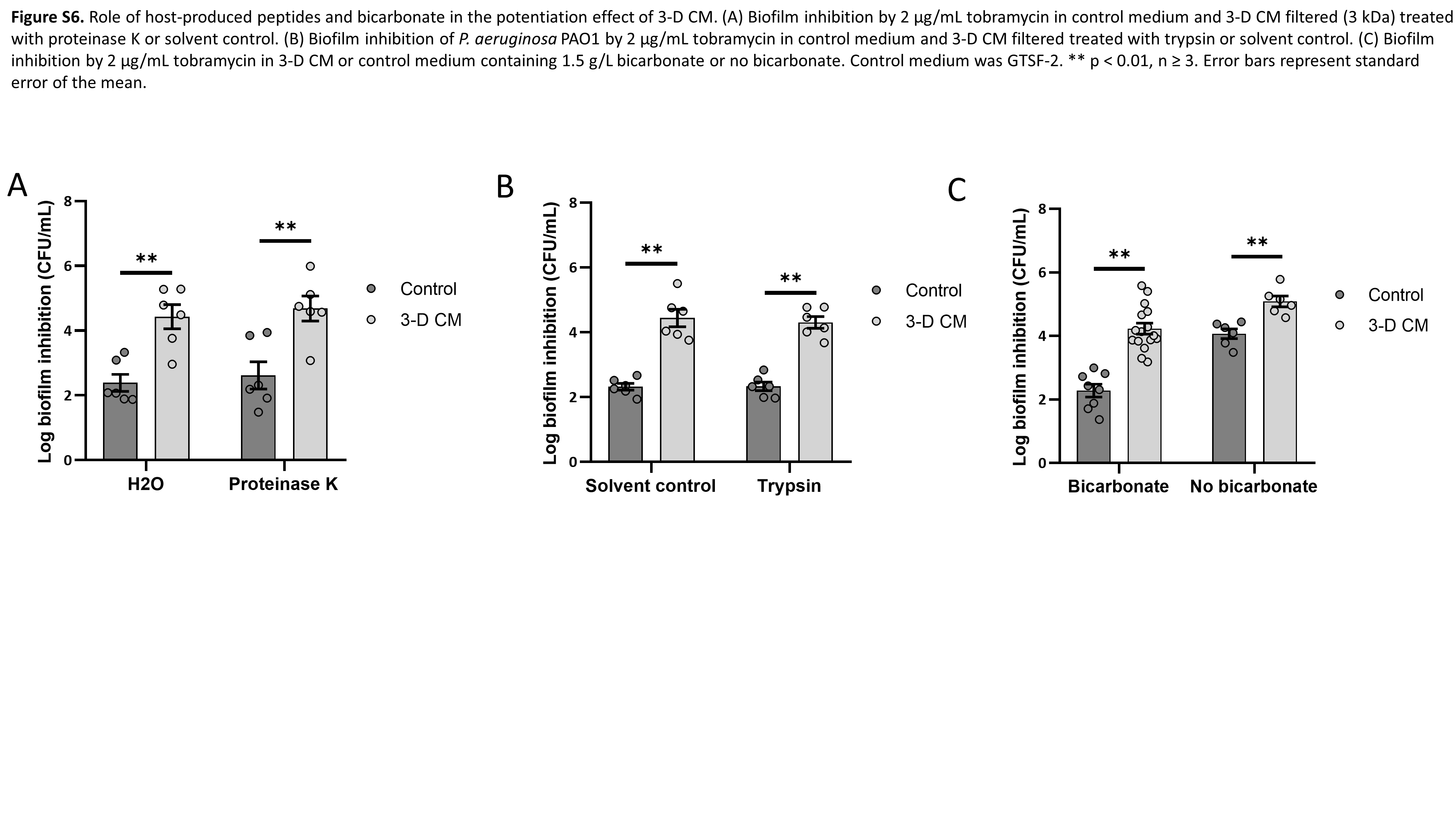

Supplement: S6 Fig — (A) Biofilm inhibition by 2 μg/mL tobramycin in control medium and 3-D CM filtered (3 kDa) treated with proteinase K or solvent control. (B) Biofilm inhibition of P. aeruginosa PAO1 by 2 μg/mL tobramycin in control medium and 3-D CM filtered treated with trypsin or solvent control. (C) Biofilm inhibition by 2 μg/mL tobramycin in 3-D CM or control medium containing 1.5 g/L bicarbonate or no bicarbonate. Control medium was GTSF-2. ** p < 0.01, n ≥ 3. Error bars represent standard error of the mean. (TIF) [file ppat.1007697.s006.TIF]

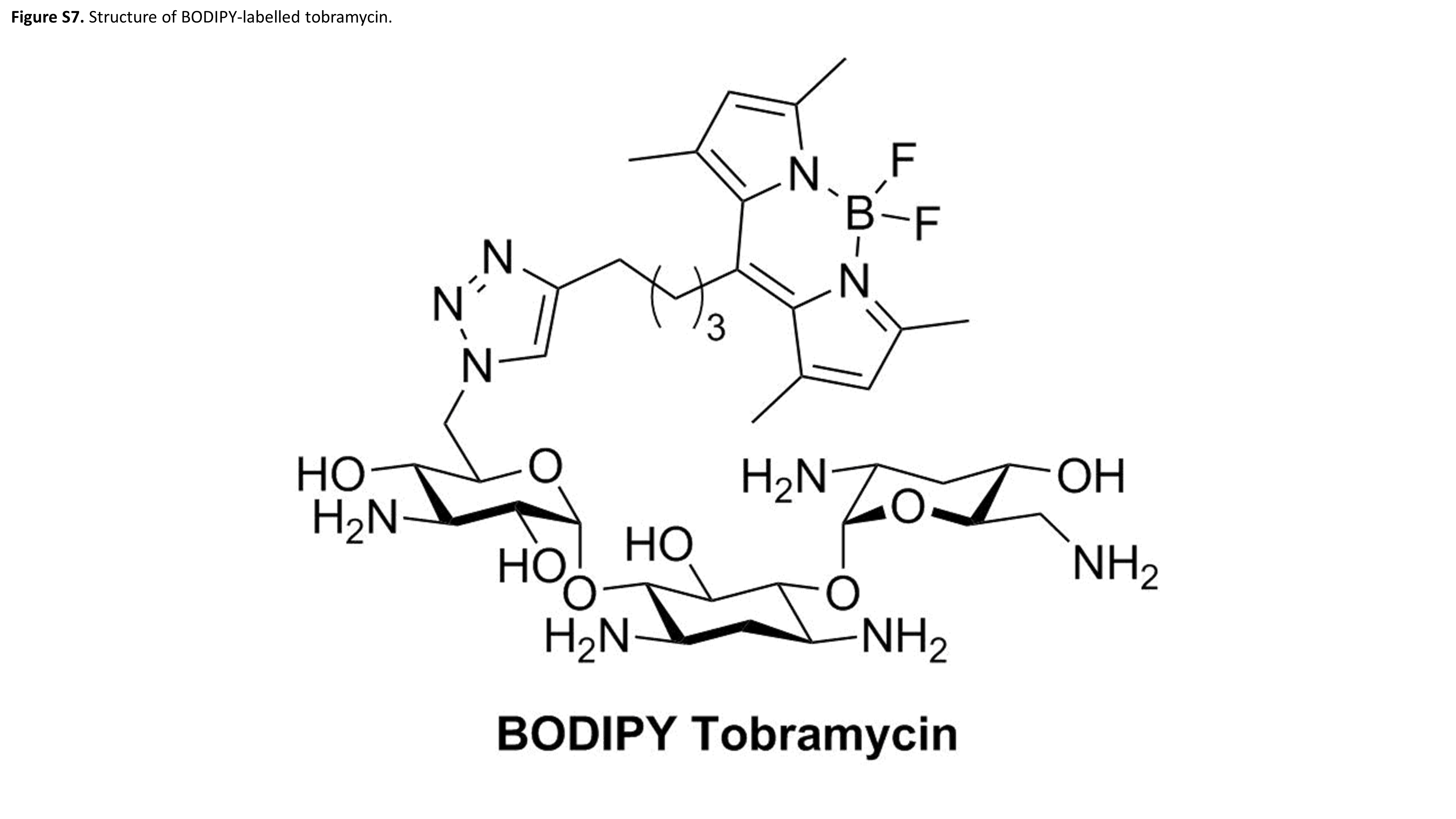

Supplement: S7 Fig — (TIF) [file ppat.1007697.s007.TIF]

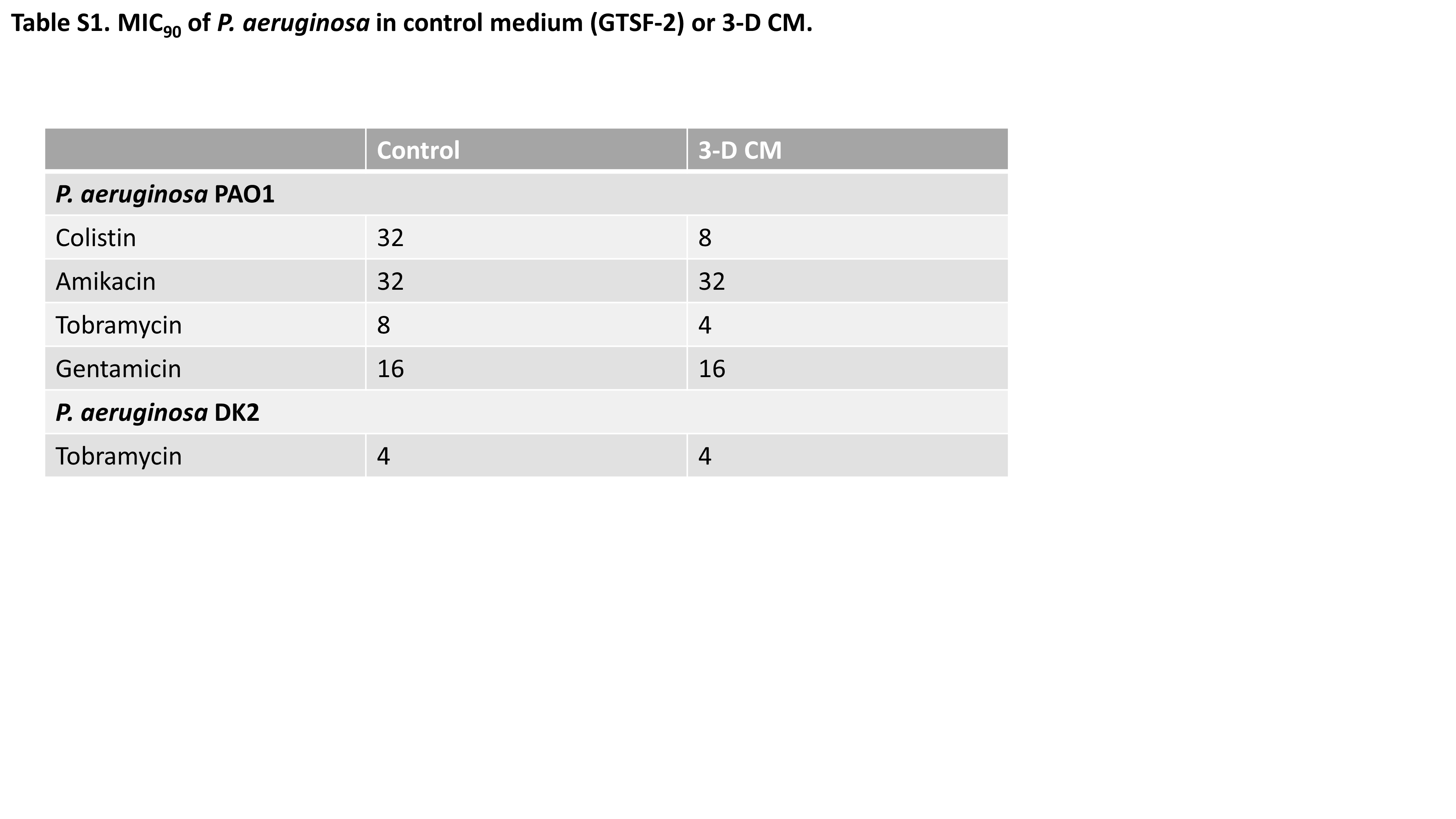

Supplement: S1 Table — (TIF) [file ppat.1007697.s008.TIF]
