# Supplementary material for: Host metabolites stimulate the bacterial proton motive force to enhance the activity of aminoglycoside antibiotics
Source: PLoS Pathog. 2019 Apr 29;15(4):e1007697. doi: 10.1371/journal.ppat.1007697 (PMC6508747; doi:10.1371/journal.ppat.1007697)
Supplement: S1 Text — Schematic overview of the BODIPY-tobramycin synthesis process and NMR spectra for synthesized BODIPY-tobramycin. (PDF) [file ppat.1007697.s010.pdf]

Supporting information: Synthesis of BODIPY-tobramycin

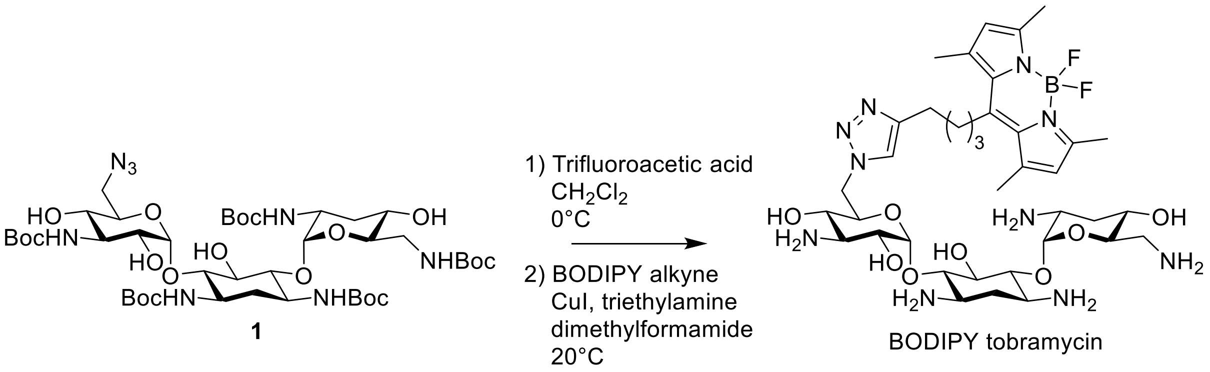

Figure 1. Overview of BODIPY-tobramycin synthesis

A solution of 6”-azido-(penta-NHBoc)-tobramycin (Fig.1 (**1**)) [1] (0.15 mmol, 150 mg) in CH_2Cl_2 (5 mL) was cooled on ice to 0°C. Trifluoroacetic acid (5mL) was added. After stirring for 2h at 0°C, the solution was concentrated *in vacuo* and the residue was dissolved in dimethylformamide (2.5 mL). The following compounds were added to the solution: CuI (29 mg, 0.15 mmol, 1 eq), diisopropylethylamine (50 µL, 0.30 mmol, 2 eq) and Bodipy alkyne [2] (50 mg, 0.15 mmol, 1 eq). The reaction mixture was stirred for 4 h at room temperature and then the solvent was evaporated. Purification with preparative thin layer chromatography (CH_2Cl_2/MeOH/NH_4OH) afforded the BODIPY Tobramycin (102 mg, 0.10 mmol 68 %) as a deep orange Electrospray ionization high resolution mass spectrometry (ESI-HRMS) for $C_{37}H_{59}BF_2N_{10}O_8$ [M+H] found, 821.4665; calcd, 821.4651. ESI-HRMS spectra were measured with a Waters LCT Premier XE Mass spectrometer calibrated using leucine enkephalin as an external standard. Samples were infused in water/acetonitrile/formic acid mixture (1:1:0.001 v/v/v) at 100 µl/min. The structure of BODIPY-tobramycin was confirmed using Nuclear magnetic resonance (NMR) spectroscopy (BRUKER 700MHz Avance II NMR spectrometer at 298K) (**Table 1**).

Table 1. NMR spectra for synthesized BODIPY-tobramcyin

NMR:	***DMSO-d6***	**700/176MHz**			
	^{1}H	^{13}C		^{1}H	^{13}C
Ring A			***Ring C***		
1	*4.80*	*100.36*	***1***	*4.94*	*99.53*
2	*2.75*	*49.26*	***2***	*3.06*	*72.08*
3	*1.42*	*37.12*	***3***	*2.87*	*55.07*
	1.82		***4***	*2.80*	*70.71*
4	*3.17*	*65.97*	***5***	*4.21*	*69.69*
5	*3.44*	*73.27*	***6***	*4.39*	*50.35*
6	*2.57*	*42.15*		*4.49*	
	2.86		***BODIPY***		
Ring B			***1/12***	*2.38*	*13.91*
1	*2.98*	*86.08*	***2/4/9/11***		*140.7*
2	*3.29*	*74.06*	***5/13***	*2.37*	*15.82*
3	*2.92*	*89.7*	***3/10***	*6.21*	*121.43*
4	*2.54*	*49.65*	***15***	*1.63*	*30.54*
5	*0.99*	*38.39*	***16***	*1.81*	*29.18*
	1.77		***17***	*2.68*	*24.49*

6	*2.56*	*51.44*	***14***	*2.95*	*27.43*
			19	*7.86*	*122.78*
			6/8		*130.6*
			7		*152.9*
			18		*146.8*

1. Disney, M.D. and O.J. Barrett, *An aminoglycoside microarray platform for directly monitoring and studying antibiotic resistance.* Biochemistry, 2007. **46**(40): p. 11223-11230.
2. Verdoes, M.; Hillaert, U.; Florea, B.I.; Sae-Heng, M.; Risseeuw, M.D.P.; Filippov, D.V.; van der Marel, G.A.; Overkleeft, H.S. *Acetylene functionalized BODIPY dyes and their application in the synthesis of activity based proteasome probes.* Bioorganic and Medicinal Chemistry Letters*,* 2007*,* **17**(22): p. 6169-6171.
